# Altered chromatin compaction and histone methylation drive non-additive gene expression in an interspecific *Arabidopsis* hybrid

**DOI:** 10.1186/s13059-017-1281-4

**Published:** 2017-08-22

**Authors:** Wangsheng Zhu, Bo Hu, Claude Becker, Ezgi Süheyla Doğan, Kenneth Wayne Berendzen, Detlef Weigel, Chang Liu

**Affiliations:** 1 Department of Molecular Biology, Max Planck Institute for Developmental Biology, Tübingen, 72076 Germany; 2 Center for Plant Molecular Biology (ZMBP), University of Tübingen, Auf der Morgenstelle 32, Tübingen, 72076 Germany; 3 Present Address: Gregor Mendel Institute, Austrian Academy of Sciences, Dr. Bohr-Gasse 3, A-1030, Vienna, Austria

**Keywords:** Interspecific hybridization, Epigenome, Chromatin structure, Non-additive gene expression, Hi-C, *Arabidopsis*

## Abstract

**Background:**

The merging of two diverged genomes can result in hybrid offspring that phenotypically differ greatly from both parents. In plants, interspecific hybridization plays important roles in evolution and speciation. In addition, many agricultural and horticultural species are derived from interspecific hybridization. However, the detailed mechanisms responsible for non-additive phenotypic novelty in hybrids remain elusive.

**Results:**

In an interspecific hybrid between *Arabidopsis thaliana* and *A. lyrata*, the vast majority of genes that become upregulated or downregulated relative to the parents originate from *A. thaliana*. Among all differentially expressed *A. thaliana* genes, the majority is downregulated in the hybrid. To understand why parental origin affects gene expression in this system, we compare chromatin packing patterns and epigenomic landscapes in the hybrid and parents. We find that the chromatin of *A. thaliana*, but not that of *A. lyrata*, becomes more compact in the hybrid. Parental patterns of DNA methylation and H3K27me3 deposition are mostly unaltered in the hybrid, with the exception of higher CHH DNA methylation in transposon-rich regions. However, *A. thaliana* genes enriched for the H3K27me3 mark are particularly likely to differ in expression between the hybrid and parent.

**Conclusions:**

It has long been suspected that genome-scale properties cause the differential responses of genes from one or the other parent to hybridization. Our work links global chromatin compactness and H3K27me3 histone modification to global differences in gene expression in an interspecific *Arabidopsis* hybrid.

**Electronic supplementary material:**

The online version of this article (doi:10.1186/s13059-017-1281-4) contains supplementary material, which is available to authorized users.

## Background

Interspecific hybridization is a common phenomenon in plants, as already recognized by Charles Darwin (reviewed in [1]). Hybrids are of interest to both evolutionary biologists and breeders, because they often show non-additive phenotypes, being either considerably more or less fit than the parents (reviewed in [2–7]). Although genetic distance clearly plays a role in dictating the extent of non-additive phenotypes, the relationship between whole-genome divergence and hybrid vigor or weakness is complex [8–11].

Interspecific hybridization can be regarded as an invasion of each parental genome by foreign genetic elements and this can lead to immediate and extensive genomic modifications [12–19]. “Transcriptome shocks,” characterized by dramatic changes in gene expression, have been widely observed after interspecific hybridizations of plants and may contribute to the evolutionary success of emerging hybrids [17, 18, 20, 21]. The changes in gene expression after hybridization have been, for example, attributed to either structural changes in the genome (e.g. resulting from loss of parental genomic fragments or mobilization of transposable elements) [16, 22, 23], complementation of recessive alleles [24–26], or epigenetic modifications [27–32]. In interspecific *Arabidopsis* hybrids, there can be striking asymmetry in expression changes of alleles derived from either parent, which has been linked to certain epigenetic chromatin marks [33, 34].

Interspecific hybridization and introgression are not uncommon in *Arabidopsis* [35] and several natural hybrids have been reported in this genus: *A. suecica* is an allopolyploid hybrid between *A. thaliana* and *A. arenosa* [36]; *A. kamchatica* is a hybrid between Siberian *A. lyrata* ssp. *petraea* and *A. halleri* [37, 38]; and extensive gene flow has been observed throughout the genus [39]. The existence of natural *Arabidopsis* hybrids stimulated early on work to understand genome-wide consequences of interspecific hybridization, including genome instability and gene expression, in such hybrids [13, 27, 40, 41]. One study reported that when genes were differentially expressed in an *A. thaliana* x *lyrata* hybrid, it was almost always biased towards higher expression of the *A. lyrata* allele [27]. Another study reported on *A. thaliana* x *arenosa* allopolyploids. Here, the vast majority of genes that were underexpressed in the hybrid relative to the mid-parental value were ones that were more highly expressed in the *A. thaliana* parent [13]. The molecular basis for these effects remains unknown.

Here, we relate genome-wide changes in gene expression in an *A. thaliana* x *lyrata* hybrid to changes in chromatin packing and epigenetic marks. We show that *A. thaliana*-derived genes are much more likely to change in expression than *A. lyrata* genes and that these are predominantly downregulated. Two important epigenetic marks, DNA methylation and H3K27me3, are mostly faithfully inherited in the F_1_ hybrid, except for transposon-rich regions which acquired elevated CHH DNA methylation. In contrast, dramatic differences were seen in chromatin compactness, with the *A. thaliana*-derived chromosomes becoming much more compact in the hybrid. In addition, among all *A. thaliana* genes, those with H3K27me3 were most likely to vary in expression between *A. thaliana* and the hybrid.

## Results

### Expression changes primarily in *A. thaliana* genes after interspecific hybridization

We generated *A. thaliana* var. Col-0 x *A. lyrata* var. MN47 F_1_ hybrid plants with *A. thaliana* as the maternal parent. A modified ovule rescue method [42] was applied to recover F_1_ hybrid plants. As reported before for interspecific hybrids of *A. thaliana* and *A. lyrata*, the hybrid plants were larger than either parent before flowering, with leaf growth rate and leaf lamina color and thickness being more similar to *A. lyrata* [42, 43].

**Fig. 1:**
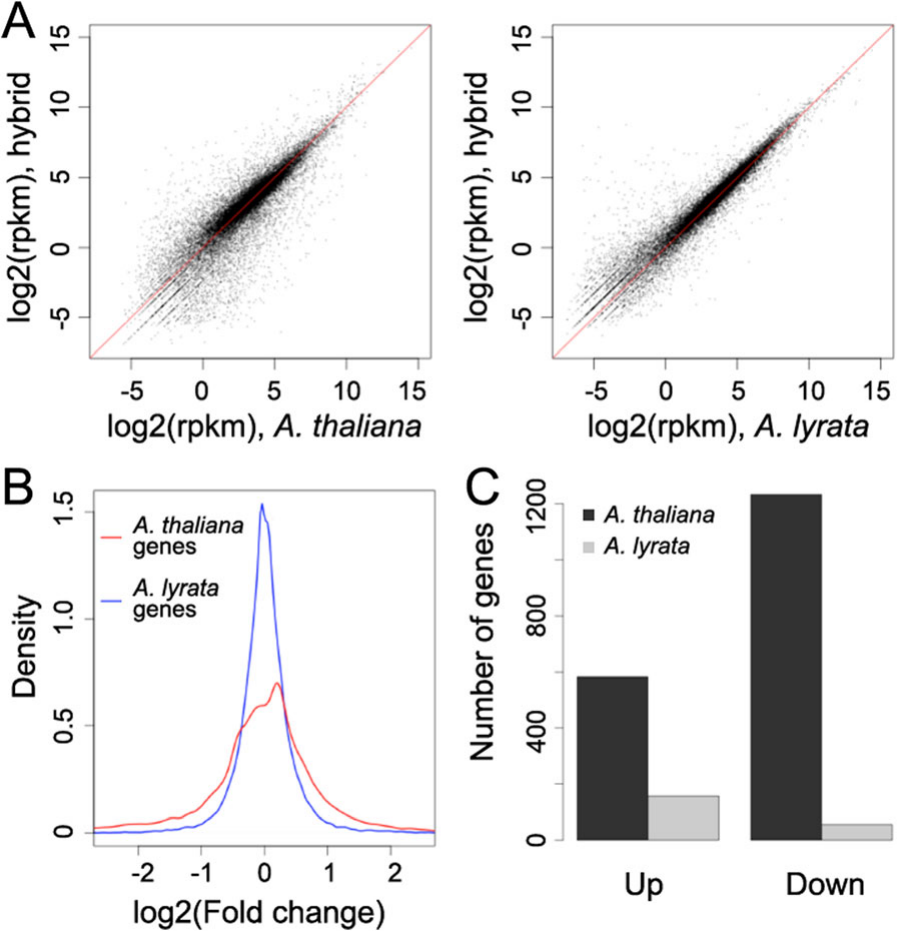
Gene expression change in hybrid plants. **a** Distribution of expression levels of *A. thaliana* (*left*) and *A. lyrata* (*right*) genes in hybrid and parents. Only genes with detectable transcripts (rpkm > 0) are included. *rpkm* reads per kilobase per million mapped reads. **b** Distribution of gene expression changes. **c** Upregulated and downregulated genes in the hybrid

To compare transcriptomes in parents and hybrid, we performed RNA-sequencing (RNA-seq) analyses by mapping RNA-seq reads to a synthetic genome consisting of both the *A. thaliana* and *A. lyrata* reference genomes, retaining only uniquely mapped reads. We then assessed both relative expression of *A. thaliana* and *A. lyrata* orthologs in the hybrid, as well as expression of these genes compared with their corresponding parent. Consistent with an earlier report on hybrid plants derived from different *A. thaliana* and *A. lyrata* accessions as parents [27], there was a systematic shift towards *A. lyrata* alleles being more highly expressed in the hybrid than the corresponding *A. thaliana* alleles (Additional file 1: Figure S1A, *p* < 2.2 × 10^–16^ with a Wilcoxon–Mann–Whitney test). However, the global expression profile of *A. thaliana* genes in the hybrid deviated much more from the *A. thaliana* parent than was the case for the *A. lyrata* genes (Fig. 1a and b). The higher expression stability of *A. lyrata* genes in the hybrid together with the higher overall expression levels likely explains the *A. lyrata*-like leaf morphology of the hybrid.

Compared with the *A. thaliana* parent, we identified 583 upregulated and 1233 downregulated *A. thaliana* genes in the hybrid; in contrast, only 156 and 55 genes from the *A. lyrata* genome were upregulated and downregulated, respectively (Fig. 1c and Additional file 2: Table S1). The upregulated or downregulated *A. thaliana* genes were distributed across all chromosomes (Additional file 1: Figures S1B and S1C). That *A. thaliana* genes are much more likely to become downregulated than upregulated in the hybrid has also been found in interspecific crosses between *A. thaliana* and *A. arenosa*, a close relative to *A. lyrata* [44], with hybrid plants resembling mostly the *A. arenosa* parent [13]. Thus, the altered expression specifically of the *A. thaliana* genes in interspecific hybrids appears to be a property of *A. thaliana* chromosomes [14].

Gene ontology (GO) analysis of the 1233 downregulated genes revealed a highly significant enrichment of genes associated with oxidative stress-response, metabolic processes, and cell wall organization, while upregulated genes were enriched for developmental processes related genes (Additional file 3: Table S2).

### Increased CHH methylation in the hybrid

The systematically altered expression of *A. thaliana* genes in the hybrid suggested that this might be due to global epigenetic changes of the *A. thaliana* chromosomes. DNA methylation plays a pivotal role in regulating chromatin activities and can affect gene expression (reviewed in [45–47]). Due to a nearly fivefold higher TE density on the *A. lyrata* chromosome arms, they are on average much more methylated than those of *A. thaliana* [48, 49]. Global DNA methylation is often reprogrammed in hybrids, which can be viewed as an “epigenetic shock” from the combination of distinct parental epigenomes, even though changes of methylation in F_1_ plants might not directly result in altered gene expression [50].

**Fig. 2:**
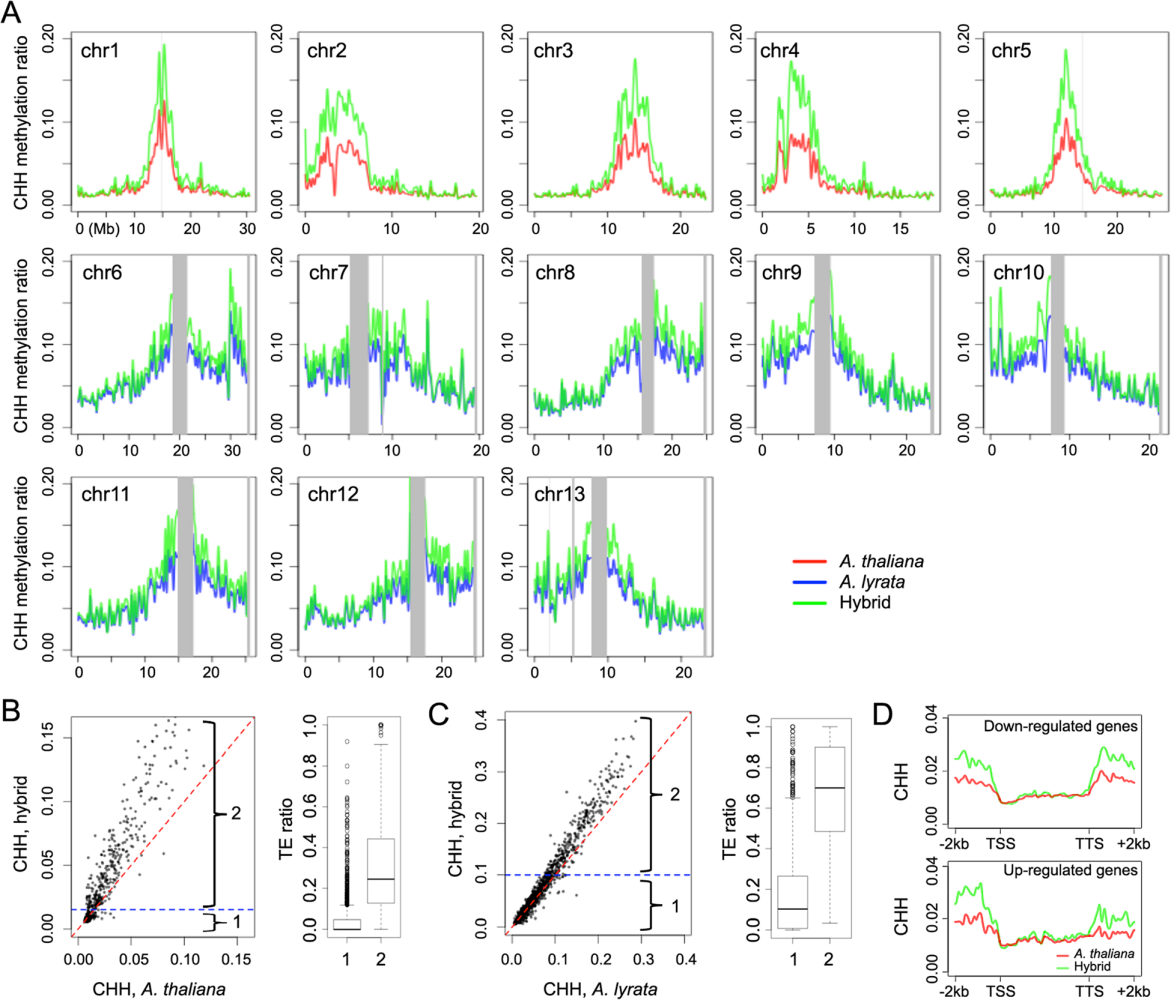
CHH DNA methylation in hybrid and parents. **a** DNA methylation along chromosomes, calculated from non-overlapping 50 kb bins. *Gray columns* indicate masked regions represented as Ns in the reference genome. **b**, **c** Comparison of CHH methylation for the first 10 Mb of *A. thaliana* chromosome 1 (**b**) and *A. lyrata* chromosome 1 (**c**). For each panel, the *plot* on the *left* is calculated from 5-kb bins, which were divided into two groups based on an arbitrarily set cutoff (*blue dash line*). Groups 1 and 2 were defined as low and high CHH methylation. The *box plots* on the right indicate the fractions of annotated transposable elements (TE) (from [49]) in group 1 and 2 bins. **d** Comparison of CHH DNA methylation between *A. thaliana* and the hybrid over genes that are downregulated (*upper panel*) or upregulated (*lower panel*) in the hybrid. In each plot, genes are scaled so that their transcriptional start sites (TSSs) and transcriptional termination sites (TTSs) are aligned. The *y-axis* indicates average methylation, calculated from non-overlapping 100-bp windows

At a chromosomal level, we found that CHH methylation of the *A. thaliana* chromosomes increased in the hybrid, particularly in the centromeric and pericentromeric regions (Fig. 2a). The same regions showed only small increases in CHG methylation and CG methylation remained largely unchanged (Additional file 1: Figure S2). CHH methylation of the *A. lyrata* chromosomes was slightly increased in the hybrid, with a slight decrease of both CG and CHG methylation (Fig. 2a and Additional file 1: Figure S2). Although the *A. lyrata* centromeric regions were not accessible to analysis, adjacent regions experienced the largest increases in CHH methylation (Fig. 2), leading us to speculate that this trend might be even stronger for the pericentromeres themselves. The higher increment of CHH methylation around pericentromeres might be related to the higher density of TEs in these regions. In agreement, we found that CHH methylation increase correlated with TE density along the chromosome arms, indicating a TE-specific increase of CHH methylation in the hybrid (Fig. 2b and c).

CHH methylation at TEs is controlled by two partially overlapping pathways, with DRM1 (DOMAINS REARRANGED METHYLASE 1) and DRM2 being key factors responsible for CHH methylation and further contribution by an RdDM-independent CHROMOMETHYLASE 2 (CMT2)-dependent pathway [51–55]. Consistent with TEs in the pericentromere cores being mainly targeted by the RdDM-independent pathway (reviewed by [56]), *A. thaliana* genomic regions with elevated CHH methylation in the hybrid were those that have been reported to be preferentially sensitive to the loss of *CMT2*, but not *DRM1/2* (Additional file 1: Figure S3A) [51, 52]. We observed a similar pattern, albeit with a weaker tendency, along the chromosome arms (Additional file 1: Figure S3B). Methylome changes, however, could apparently not explain the changes in expression of protein-coding genes in the hybrid (Fig. 2d and Additional file 1: Figure S4).

### Increased compactness of *A. thaliana* chromatin in the hybrid

**Fig. 3:**
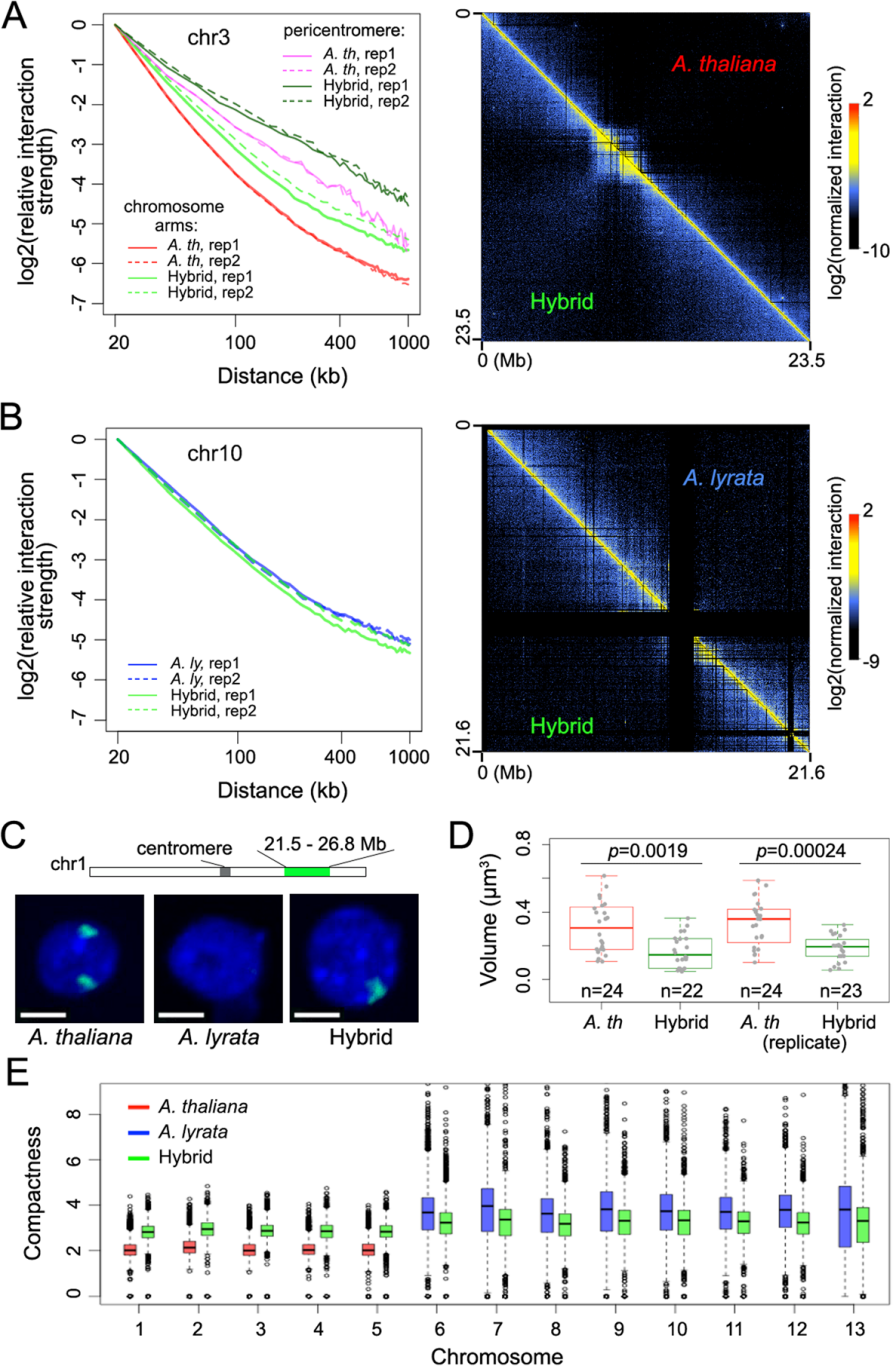
Changes of chromosomal level chromatin compactness. **a**, **b** Comparisons of *A. thaliana* chromosome 3 and *A. lyrata* chromosome 5 (chromosome 10 in the synthetic hybrid reference genome) are shown in (**a**) and (**b**), respectively. For each *panel*, data from each biological replicate are shown on the *left* and the Hi-C maps from combined replicates are shown on the *right*. The *plot* on the *left* shows interaction decay exponents, calculated from Hi-C maps at 20-kb resolution, which are shown on the *right*. For each curve, the contact frequency at 20-kb distance is set to 1. To make colors in the two Hi-C maps comparable, values in each normalized Hi-C map were divided by the average value indicative of interactions between bins 20 kb apart. **c**, **d** FISH experiments. **c** A 5.3-Mb region (highlighted in *green* in the sketch) of the *A. thaliana* genome can be specifically detected with a BAC probe mixture. Scale bars, 2 μm. **d** Space volume occupied by the probed *A. thaliana* genomic region in the hybrid and the parent nuclei. *p* values mean results of Wilcoxon–Mann–Whitney tests. **e** Analysis of chromatin compactness derived from Hi-C maps at 5-kb resolution. Pericentromeric regions in *A. thaliana* are excluded

DNA methylation is highly correlated with tightly packed heterochromatin (reviewed in [57]). The substantial increase of CHH methylation associated with the *A. thaliana* pericentromeric regions prompted us to ask whether the packing patterns of chromatin in hybrid and parents differed accordingly. To this end, we employed a genome-wide Chromatin Conformation Capture approach, Hi-C [58], to compare chromatin packing. We adopted an in situ Hi-C protocol that better preserves chromatin folding compared to the regular “dilution” Hi-C method [59–61]. After stringent read mapping and filtering, we obtained about 20 million, 38 million, and 85 million of true Hi-C reads from *A. thaliana*, *A. lyrata*, and the hybrid, respectively (Additional file 4: Table S3). The normalized Hi-C maps showed strong signals along their diagonals, resulting from stochastic contacts between sequences close to each other in the linear genomes (Additional file 1: Figure S5). Visual inspection indicated that at a chromosomal level, the *A. thaliana* but not *A. lyrata* chromatin had more intra-chromosomal contacts over long distances in the hybrid (Fig. 3a and b). This pattern was confirmed by comparing the power-law decay curves of intra-chromosomal interaction strength with genomic distance (Fig. 3a and b). Notably, both the pericentromeres and chromosome arms of *A. thaliana* showed less steep decay slopes in the hybrid compared to the parent (Fig. 3a). A possible scenario explaining these patterns might be that hybridization caused the *A. thaliana* chromosomes to become more compact and occupy smaller nuclear volumes, thereby increasing the likelihood and thus strength of long-distance chromatin interactions. To test this idea, we performed fluorescence in situ hybridization (FISH) with a pool of probes covering a 5.3-Mb genomic region (21.5 Mb to 26.8 Mb) in a non-pericentromeric region of *A. thaliana* chromosome 1 (see “Methods”). These probes did not produce a signal when hybridized to the *A. lyrata* genome (Fig. 3c). By calculating the volume of FISH signals (see “Methods”), we found that the signal occupied significantly less space in the hybrid nuclei than it did in the parent (Fig. 3d), indicating an increase in chromatin compactness of *A. thaliana* chromosomes in the hybrid.

Similarly, we observed a higher degree of *A. thaliana* chromatin compactness at local levels with Hi-C. By comparing the distribution of Hi-C reads as a function of genomic distance, we found that compared to the parent, *A. thaliana*, but not *A. lyrata*, chromosomes in the hybrid engaged in more long-range interactions (Additional file 1: Figure S6). From Hi-C maps normalized at 5-kb resolution, we approximated the chromatin compactness of 5-kb regions by calculating the strength of their interactions with surrounding regions located 10–50 kb away (see “Methods”). Overall, in the parents, chromatin was more compact in *A. lyrata* than in *A. thaliana* (Fig. 3e, compare red and blue boxplots). In the hybrid, the *A. thaliana* chromatin became more compact, whereas the *A. lyrata* chromatin became moderately less compact (Fig. 3e). Nonetheless, in the hybrid, *A. lyrata* chromatin still had higher compactness than *A. thaliana* chromatin (Fig. 3e, compare green boxplots). We next examined potential connections between DNA methylation and chromatin packing in the hybrid and its parents. As expected, in all three genotypes, highly methylated chromatin regions had less negative interaction decay exponents (Additional file 1: Figure S7). The interaction decay exponents describe how fast chromatin interaction strengths drop with increasing genomic distance; as expected, heterochromatin, which is often tightly packed, has less negative values than euchromatin (see “Methods”). In the hybrid, *A. thaliana* chromatin was shifted towards less negative interaction decay exponents, making it more similar to *A. lyrata* chromatin (Additional file 1: Figure S7, see the last column). Overall, for all three types of plants, the more DNA methylation there is in a genomic region, the more highly compacted the chromatin is.

### Relationship between chromatin compactness, DNA methylation, and gene expression changes in the hybrid

**Fig. 4:**
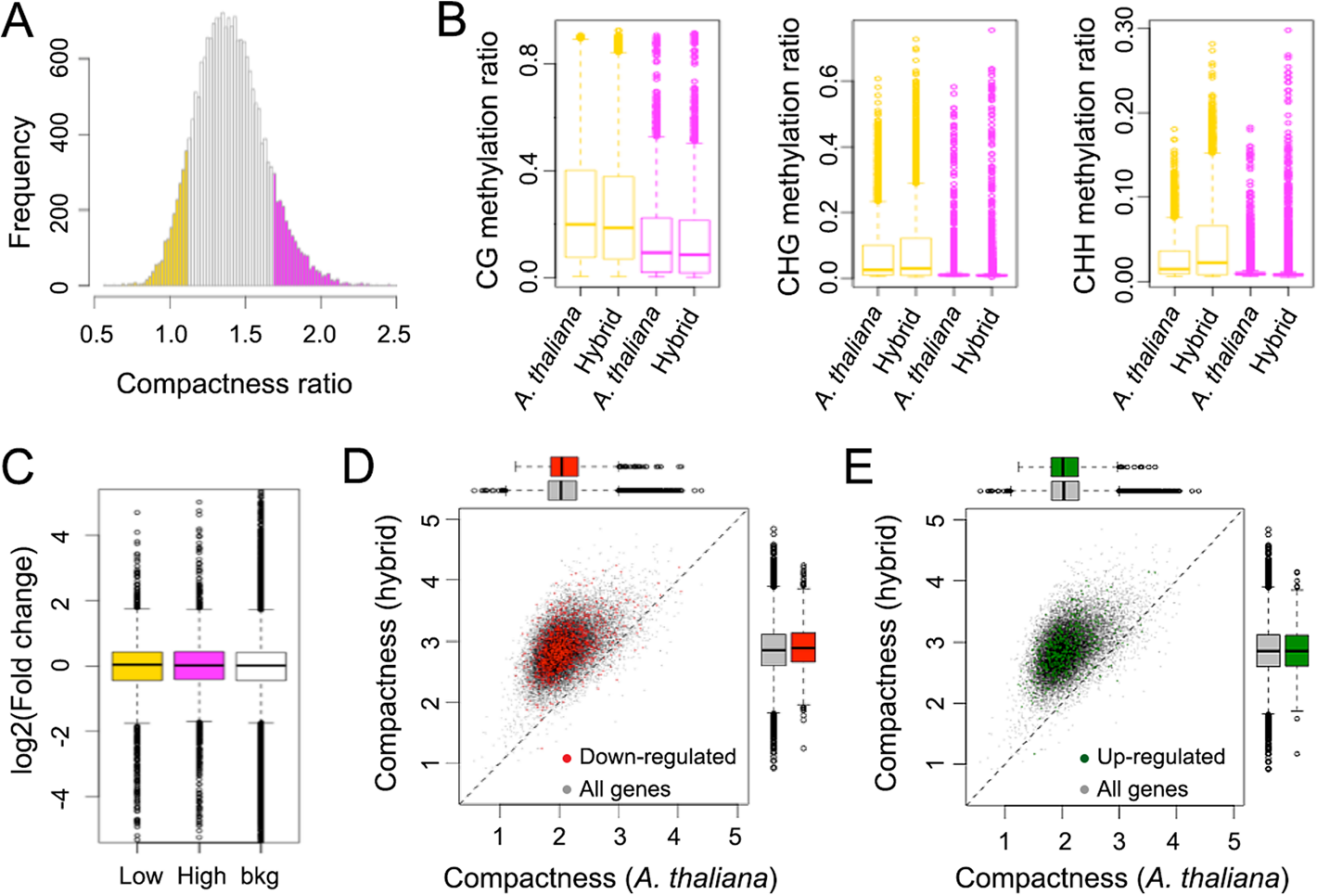
Association between chromatin compactness, DNA methylation, and gene expression. **a** Distribution of *A. thaliana* chromatin compactness ratios between the hybrid and parent, calculated in 5-kb bins. Areas highlighted in *yellow* and *magenta* indicate the bottom and top 10% bins, respectively. **b** DNA methylation of bins highlighted in (**a**). **c** Distribution of gene expression ratios between hybrid and *A. thaliana* parent in regions highlighted in (**a**). Every gene is assigned to a 5-kb bin based on its TSS. *Bkg* background consisting of all genes. All comparisons have *p* values > 0.05 from Wilcoxon–Mann–Whitney tests. **d**, **e** Relationship between chromatin compactness and differentially expressed genes. Bins of 5 kb are colored *red* (**d**) or *green* (**e**) if there is at least one upregulated or downregulated gene located in them. For both panels, all pairs of comparisons have *p* values > 0.05 from Wilcoxon–Mann–Whitney tests

The chromatin packing effects were much more extensive than the DNA methylation changes, suggesting that altered DNA methylation is not the cause, or at least not the only cause, for changes in chromatin compaction. We used ratios of the *A. thaliana* chromatin compactness in the hybrid over its parent as an indicator of chromatin compaction. Selecting the top and bottom 10% (Fig. 4a), we found that the regions with lowest compactness ratios tended to have higher DNA methylation in all sequence contexts (Fig. 4b, compare yellow and magenta box plots), which could not be explained by the differences of methylation between the hybrid and parents (Fig. 4b, compare box plots with the same color). Thus, the systematic increase of *A. thaliana* chromatin compactness in the hybrid was likely due to factor(s) other than DNA methylation.

Changes in chromatin compactness might affect its accessibility to transcription and chromatin remodeling factors, which could ultimately result in changes in gene expression levels [62, 63]. Given the fact that hybridization caused the *A. thaliana* chromatin to generally become more compact, we next assessed whether this was correlated with changes in the *A. thaliana* transcriptome (Fig. 1). Comparison of changes in gene expression in regions with the highest and lowest compactness ratios did not reveal any differences between the two classes (Fig. 4c). Similarly, we did not observe upregulated or downregulated genes to be more or less likely located in regions with low or high chromatin compactness ratios (Fig. 4d and e). Thus, changes in *A. thaliana* chromatin compactness in the hybrid do not seem to be directly linked to changes in gene expression.

On the other hand, we observed that intra- and inter-chromosomal interactions among *A. thaliana* heterochromatin islands, known as KEEs or IHIs [64, 65], tended to be weaker in the hybrid (Additional file 1: Figure S8). Instead, KEE/IHI chromatin interacted more strongly with directly flanking sequences (Additional file 1: Figure S8B), which are more euchromatic [64]. We therefore asked whether this affected the transcriptional activities of KEE/IHI regions, but found that genes located in KEEs/IHIs showed a similar profile of expression changes as the background (Additional file 1: Figure S8C). Collectively, our results suggest that after hybridization there is no close relationship between changes in chromatin compactness and gene expression on the *A. thaliana* chromosome arms.

### Enrichment of H3K27me3 in differentially expressed *A. thaliana* genes

**Fig. 5:**
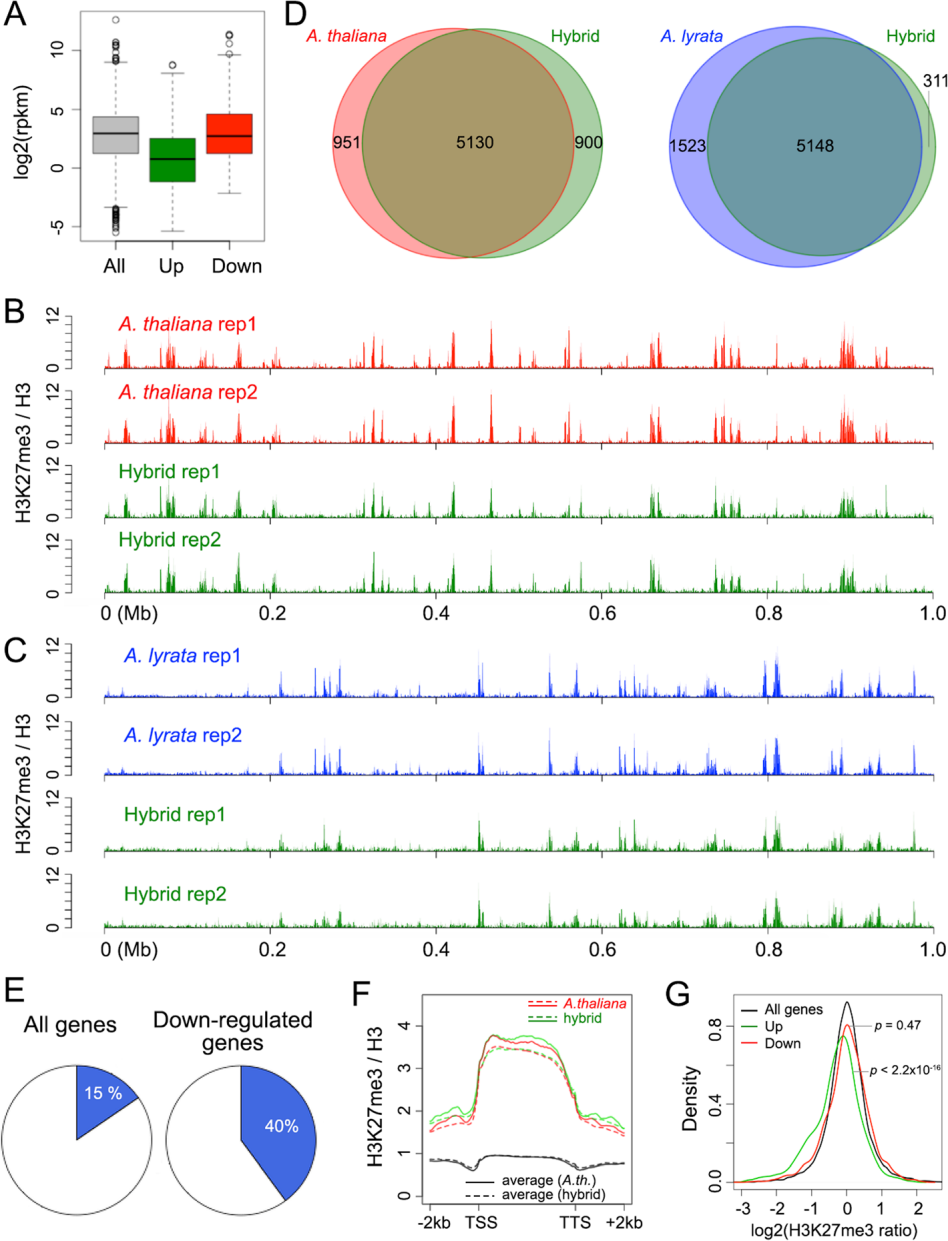
H3K27me3-marked genes are enriched among genes downregulated in the hybrid. **a** Expression in *A. thaliana* of genes upregulated or downregulated in the hybrid. *Green* and *red boxplots* indicate genes upregulated and downregulated in the hybrid. **b**, **c** Distribution of H3K27me3 marks along a 1-Mb region on the *A. thaliana* chromosome 3 (**b**) and the *A. lyrata* chromosome 5 (chromosome 10 in the synthetic hybrid reference genome) (**c**), plotted as the ratio of normalized coverage between H3K27me3 and H3 ChIP-seq with a 100-bp window setting. **d** Number of genes marked by H3K27me3 in different genotypes. A gene is considered marked with H3K27me3 if more than half of its transcribed region overlaps with H3K27me3 enrichment peaks. **e** Fraction of H3K27me3-marked genes shared by *A. thaliana* and the hybrid. The *pie chart* on the *left* refers to all *A. thaliana* genes, the one on the *right* to genes downregulated in the hybrid. Genes marked with H3K27me3 both in the hybrid and parent are colored *blue*. **f** Comparison of the H3K27me3 signals over *A. thaliana* gene bodies in hybrid and parent. For the parent, the *red dashed curve* indicates the average of all H3K27me3-marked genes and the *red solid curve* indicates the average of the subset downregulated in the hybrid and marked with H3K27me3 in both hybrid and parent. The *green curves* refer to the same sets in the hybrid. *Black solid* and *dash curves* indicate the average of all *A. thaliana* genes in the hybrid and parent. **g** Ratios of H3K27me3 signals in the hybrid over the parent in *A. thaliana* gene bodies. Upregulated or downregulated genes are the same as in (**a**). *p* values mean Wilcoxon–Mann–Whitney tests

Next, we sought to analyze whether changes in expression of *A. thaliana* genes in the hybrid were associated with genomic or epigenetic features of individual genes. Compared to the profile of all genes, upregulated or downregulated *A. thaliana* genes did not noticeably differ in length, exon number, and exon or intron size (Additional file 1: Figure S9). By making use of a previous analysis of the *A. thaliana* seedling epigenome based on a series of histone marks and variants [66], we found that both upregulated and downregulated *A. thaliana* genes could be differentiated from the genomic average of several marks in the parent, with the most prominent differences in H3K27me3, H3K36me3, H2A, and H2A.Z (Additional file 1: Figure S10). That the histone variant H2A.Z in gene bodies is associated with genes that are particularly sensitive to environmental or developmental changes [67, 68] agrees with our finding that such genes are highly enriched among the genes differentially expressed in the hybrid (Additional file 3: Table S2). An over-representation of H3K27me3 in genes differentially expressed in the hybrid could simply be due to most of these genes being only weakly expressed in the *A. thaliana* parent, hence the increased H3K27me3 levels. This was indeed the case for genes upregulated in the hybrid (Fig. 5a). However, downregulated genes, which contributed the majority (68%, see Fig. 1c) of differentially expressed *A. thaliana* genes, did not differ from the average in terms of expression level in the parent (Fig. 5a).

To understand why genes downregulated in the hybrid were enriched for the H3K27me3 mark, we compared the H3K27me3 landscape between parents and hybrid using ChIP-seq. We found highly similar H3K27me3 patterns in chromatin of hybrid and parents (Figs. 5b and c, Additional file 1: Figure S11), similar to observations made with intra-specific hybrids of *A. thaliana* [69, 70]. There were a few exceptional loci with contrasting H3K27me3 levels in different genotypes, such as *FLOWERING LOCUS C* (*FLC*), which showed a complete loss of this mark throughout the coding region in the hybrid (Additional file 1: Figure S12). In this specific case, this was likely due to the presence of functional *FRIGIDA* (*FRI*) in the *A. lyrata* genome, activating *FLC* expression [71–73]. Most genes enriched for H3K27me3 in the parental genome maintained this mark in the hybrid (5130/6081 for *A. thaliana* genes and 5148/6671 for *A. lyrata* genes, Fig. 5d), indicating conservation of this histone mark at a global level upon hybridization. In line with our results derived from comparing differentially expressed genes with a variety of epigenetic marks (Additional file 1: Figure S10), genes marked with H3K27me3 in both the hybrid and *A. thaliana* were over-represented among genes downregulated in the hybrid (Fig. 5e). We wondered whether this downregulation was due to increased H3K27me3 deposition in the hybrid, but this was apparently not the case (Fig. 5f). Furthermore, compared to the total *A. thaliana* genes, as expected, genes that became upregulated in the hybrid showed a tendency of losing H3K27me3; however, downregulated genes did not show an increase in abundance of this histone mark in the hybrid (Fig. 5g).

### Increased expression variance of H3K27me3-marked *A. thaliana* genes in the hybrid

**Fig. 6:**
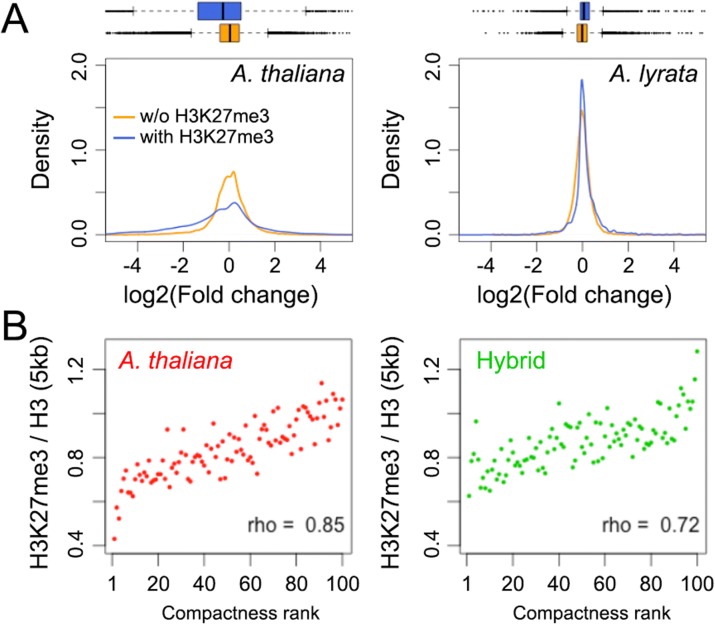
Effects of hybridization on expression of H3K27me3-marked genes. **a** Distribution of *A. thaliana* (*left*) and *A. lyrata* (*right*) genes with respect to gene expression change in the hybrid. Only genes having similar H3K27me3 enrichment in parent and hybrid are included, where genes marked with H3K27me3 in both types of plants are colored in *blue* and genes not marked by H3K27me3 in either type of plant are colored *orange*. **b** Average H3K27me3 signals in *A. thaliana* chromatin in the hybrid and the parent ranked from weakest to strongest chromatin compactness

The enrichment of the H3K27me3 mark among *A. thaliana* genes differentially expressed in the hybrid prompted us to further test whether on a genome-wide scale, *A. thaliana* genes labeled with H3K27me3 tended to have more transcriptional changes between the hybrid and parent. To this end, we analyzed genes enriched for H3K27me3 in both the hybrid and its parents (Additional file 5: Table S4). We observed parent-dependent changes of gene expression in the hybrid, with H3K27me3 marked *A. thaliana* genes, but not *A. lyrata* genes being downregulated (with *p* values < 2.2 × 10^–16^ and = 1.0 from one-sided Wilcoxon–Mann–Whitney tests, respectively) (Fig. 6a). Moreover, on the *A. thaliana* side, compared to non-H3K27me3 target genes, we observed a larger variance of gene expression among genes marked with H3K27me3; however, this difference was not observed among *A. lyrata* genes (Fig. 6a). For both the *A. thaliana* and *A. lyrata* chromatin, the chromosomal H3K27me3 landscapes were similar between hybrid and parents (Fig. 5). Thus, being enriched for H3K27me3 per se was not sufficient to explain the differences in expression changes in the two subgenomes.

The repressive histone mark H3K27me3 has recently emerged as a key factor in regulating higher order chromatin organization in plants [64, 66, 74–76]. In *A. thaliana*, H3K27me3 is required for long-range chromatin interactions among certain loci [64] and is over-represented in the promoter regions of genes that are in conformational linkage over long distances [66]. In both the hybrids and its parents, this histone mark was positively correlated with chromatin compactness (Fig. 6b and Additional file 1: Figure S13), suggesting that to a certain extent it is involved in spatial genome organization. Recent findings in animals indicate that transcriptional regulation of H3K27me3-marked genes depends on contacts within three-dimensional chromatin networks [77–80]. At a genomic level, removal of Polycomb repression not only results in deregulation of gene expression, but also diminishes chromatin interactions among these loci [77, 80, 81]. In a case study of the *Drosophila* Hox genes, mutations on one PcG-targeted Hox gene weakens the silencing of other Hox genes that interact with it [79]. Thus, among H3K27me3 marked genes located in the *A. thaliana* genome, different from those located in the *A. lyrata* genome, the ones with increased variance of gene expression between the hybrid and parent might be linked to global genome compaction, which reflects changes in local chromatin folding topology.

## Discussion

Gene expression studies of hybrids typically focus on additive versus non-additive gene expression of parent alleles, with an eye on understanding the phenomena of hybrid vigor and weakness (reviewed in [4, 82]). In this study, we focused instead on understanding how the transcriptomic readout of entire subgenomes in an interspecific hybrid differed from its parents. Our results revealed that genes differentially expressed between hybrid and parents were predominantly from the *A. thaliana* subgenome (Fig. 1). Similar to previous studies [27, 42], our attempts to generate hybrid plants with *A. lyrata* as the maternal parent were not successful. For this reason, one cannot assess how much maternal effects contribute to such transcriptome changes in hybrids. Nonetheless, our analyses point to differences in chromatin compactness and changes in chromatin compactness being an important driver of differential gene expression in the hybrid.

The mechanisms modulating higher-order chromatin organization in plants is poorly understood. It is known that chromatin regions with dense repressive epigenetic marks, such as DNA methylation and H3K27me3, are more tightly packed than those without such marks. For example, as the *A. lyrata* genome has more heterochromatic TE elements throughout its chromosomes [83], it is expected that *A. lyrata* chromatin is more compact than *A. thaliana* chromatin, both in the parent and the hybrid. Our Hi-C analyses show that this is indeed the case (Fig. 3e). However, the gain-of-compactness changes of *A. thaliana* chromosome arms in hybrids were not accompanied by increases in these repressive marks (Figs. 2 and 5). We do not know yet which factors caused this systematic change of chromatin compactness. For example, a dominant factor contributed by the *A. lyrata* genome might directly cause higher compaction of the *A. thaliana* genome. Also, we cannot exclude that having *A. thaliana* as the maternal parent plays a role in differential chromatin compaction. In the future, developing new culturing methods permitting reciprocal crosses between *A. thaliana* and *A. lyrata* can help address this question.

Generally, higher chromatin compactness is associated with lower gene expression [84], but such a correlation was not found at the level of individual genes (Figs. 4c–e). A possible explanation is that the average increase of *A. thaliana* chromatin compactness in the hybrid was not large enough to reach a tightly packed state comparable to inaccessible heterochromatin. As shown as an example in Fig. 3a, local compactness of *A. thaliana* chromosome 3 arms in the hybrid was still much lower than that of the pericentromeric regions in the parent. Nonetheless, genome-wide changes in chromatin compactness must ultimately be related to localized changes in chromatin torsion and tension, as well as chromatin conformation, all of which can influence DNA-protein and DNA-nucleosome interactions [85–89]. For these reasons, we speculate that there is a connection between the systematic changes in *A. thaliana* gene expression and chromatin compactness, although their causal relationship is not straightforward.

Genes labeled with the H3K27me3 histone mark were not only over-represented among differentially expressed *A. thaliana* genes (Fig. 5e and Additional file 1: Figure S10), but they also tended to have higher expression variance between hybrid and parents (Fig. 6a). Since the chromosome-scale H3K27me3 epigenomic landscapes of both the *A. thaliana* and *A. lyrata* genomes were basically unaltered in the hybrid (Fig. 5 and Additional file 1: Figure S11), one would like to know why *A. thaliana* H3K27me3 marked genes, but not *A. lyrata* H3K27me3 marked genes were affected in their expression in the hybrid. Because the H3K27me3 histone mark has an important role in the local recruitment of Polycomb group (PcG) proteins, it is highly relevant for long-range chromatin interactions. Several recent studies in animals have revealed that PcG proteins regulate gene expression in a three-dimensional network of chromatin contacts, where H3K27me3-marked chromatin regions that are distant along the chromosome tend to co-localize, to achieve coordinated transcriptional readouts of multiple genes [77–81]. In plants, genome-wide studies of chromatin packing have suggested that H3K27me3, along with the associated PcG proteins, shapes genome structures [64–66, 75]. One example comes from the *FLC* locus, where the two allelic copies cluster via H3K27me3-marked chromatin, with silencing mediated by PcG proteins [74]. On a genomic scale, loss of the LIKE HETEROCHROMATIN PROTEIN 1 (LHP1) protein, which binds to H3K27me3, results in alterations of both local H3K27me3 deposition and chromatin interaction patterns [76]. From a genome topology point of view, these findings have suggested that H3K27me3-targeted genes are similarly regulated in plants and animals. As discussed above, global changes in *A. thaliana* chromatin compactness were likely due to local conformational changes, which in turn may have positioned H3K27me3-marked genes into alternative regulatory environments.

Changes in compactness of the *A. thaliana* chromatin might also underlie nucleolar dominance, the selective silencing of ribosomal RNA (rRNA) genes contributed by one of the parental genomes in a hybrid [90]. In the *A. thaliana *x *lyrata* hybrid, the *A. thaliana* arrays of rRNA genes are specifically silenced [14, 91]. Being located in one of the two nucleolus organizer regions (NORs) is a prerequisite for rRNA gene silencing in the hybrid, since these genes become expressed when relocated to ectopic locations of the parental genome [91], indicating that nucleolar dominance is affected by the state of flanking chromatin. As one potential mechanism, it has been suggested that higher TE densities in regions adjacent to *NOR2* (*NOR ON CHROMOSOME 2*) compared to those adjacent to *NOR4* account for selective silencing of *NOR2* rRNA genes [92]. Because short reads cannot be mapped to the highly repetitive NOR sequences, which in any case are not properly represented in the reference genome assemblies, we could not assess their behavior in the hybrid and parents, but we speculate that the *A. thaliana* NORs became more tightly packed and less accessible in the hybrid, as did both the *A. thaliana* chromosome arms and pericentromeric regions (Fig. 3). In *A. thaliana* nuclei, NORs preferentially interact with centromeres, which collectively form discrete foci of heterochromatin called chromocenters [93]. In the hybrid, the centromere-proximal boundaries of NOR2 and NOR4 interacted with pericentromeres as strongly as in *A. thaliana* (Additional file 1: Figure S14), suggesting maintenance of the NOR-centromere complexes. Chromocenters have been observed in *A. thaliana* x *lyrata* hybrid nuclei, appearing as conspicuous DAPI-dense spots [42]. From the higher levels of CHH methylation (Fig. 2) and higher chromatin compactness near centromeres in the hybrid (Fig. 3), it follows that if NORs are parts of chromocenters in the hybrid, they are likely in a more heterochromatic environment compared to the one in *A. thaliana*, with could contribute to selective rRNA gene silencing.

It is worth noting that chromatin compactness seems to be resistant to changes in nuclear morphology. In a recent Hi-C study, neither the *A. thaliana crowded nuclei 1* (*crwn1*) nor *crwn4* mutant, which produce smaller and more spherical nuclei, showed evidence of increased chromatin compactness in chromosome arms [65, 94], suggestive of a mechanism actively modulating chromatin packing independently of the ratio of DNA and nuclear volume.

## Conclusions

In conclusion, we report that *A. thaliana*-derived genes are much more likely to change in expression than *A. lyrata*-derived genes in an interspecific hybrid and that the differentially expressed *A. thaliana* genes tended to be labeled with the H3K27m3 histone mark. In addition, compared to the parent, *A. thaliana* chromatin compactness increases in the hybrid; while the compactness of *A. lyrata* chromatin hardly changes. By providing evidence for chromosome-scale changes of chromatin folding, we reveal a new mechanism that might underlie genome-wide differences in the behavior of two subgenomes in a hybrid.

## Methods

### Plant material

*A. thaliana* accession Columbia (Col-0) and *A. lyrata* accession MN47 [83] were used to generate hybrid plants, with *A. thaliana* as maternal parent. A modified ovule rescue method [42] was used to recover F_1_ hybrid seeds. Six days after pollination, the elongating siliques were harvested and surface sterilized with 10% bleach for 20 min at room temperature. The siliques were opened under a dissecting microscope in a laminar flow cabinet and the developing seeds were transferred to half strength Murashige & Skoog (MS) medium supplemented with 1% sucrose and 0.3% phytagel. The medium was placed in a standard long-day plant growth chamber (23 °C, with 16 h light/8 h dark cycles) to allow the hybrid seeds to mature and germinate. Upon germination, the hybrid seedlings were transferred to soil. The presence of the *A. lyrata* genome was verified by genotyping with a pair of *A. lyrata* specific primers, 5’-CATAACTTTCGTTGTTACATC-3’ and 5’-CCGAGTTATTATGATTACTATTAGTC-3’.

For materials used in RNA-seq, bisulfite-sequencing (BS-seq), in situ Hi-C, and ChIP-seq experiments, the hybrid and the parents were grown at 23 °C in long days (16 h light/8 h dark) on soil. The aerial parts of 15-day-old *A. thaliana*, 30-day-old *A. lyrata*, and hybrid seedlings, at which age the different genotypes had similar morphologies, were harvested at Zeitgeber time 6 (ZT6: 6 h after lights on). Note that differences in life history of the different samples might have contributed to observed expression and chromatin differences. However, because the growth rates and vegetative phase lengths of these plants are different, harvesting them at the same chronological time point likely leads to even stronger biases. For example, on day 10, the aerial portion of *A. lyrata* plants only includes very little true leaf material. On the other hand, on day 30, the *A. thaliana* plants will already flower.

Two biological replicates were generated for each sample in all experiments. For BS-seq, Hi-C, and ChIP-seq, reads from replicates were combined. See Additional file 1: Figures S15–S17 for details of comparisons of replicates.

### RNA-seq library preparation and analysis

Total RNA was isolated with RNeasy Plant Mini Kit (Qiagen) and libraries were prepared according to a standard protocol (Illumina). RNA-seq data from 15-day-old Col-0 seedlings were from [95], in which plants were grown in the same growth chamber and with the same settings as used in our study. Although previous work from our lab has shown minimal expression differences in genetically identical plant cohorts grown at different times in our growth chambers [96], we cannot exclude that growth at different times contributes to the drastic differences in gene expression reported here. Reads were aligned against a synthetic reference genome, consisting of both the annotated *A. thaliana* (TAIR10) and *A. lyrata* genomes (v.1.0.24, ftp://ftp.ensemblgenomes.org/pub/plants/release-24/), using TopHat 2 with default parameters [97]. For the synthetic hybrid reference genome, the *A. lyrata* chromosomes 1 to 8 [83] were re-named as chromosomes 6 to 13. Only uniquely mapped reads were retained and processed with the GenomicAlignments package in R [98]. The resulting raw count table, which contained number of mapped reads for each gene in each sample, was used to identify differentially expressed genes with the DESeq2 package in R [99]. We used criteria of false discover rate smaller than 0.05 and fold change of log2 fold greater than 2 to call upregulated and downregulated genes. Gene ontology (GO) analysis of differentially expressed *A. thaliana* alleles was performed according to [100], where the enriched GO terms were identified with GOrilla [101] and further summarized with REViGO [102]. In parallel, this count table was also used to calculate RPKM (Reads Per Kilobase per Million mapped reads) for each gene [103].

### In situ Hi-C library preparation

Tissue fixation and nuclei extraction were performed as described [75]. For one round of in situ Hi-C library preparation, 0.5 g of homogenized tissue powder produced via grinding fixed tissue under liquid nitrogen was used.

Nuclei permeabilization, chromatin digestion, and proximity ligation treatments were adapted from [60]. The extracted nuclei were resuspended in 150 μL 0.5% SDS and split equally into three tubes. To make the nuclei more permeable, the resuspended nuclei were incubated at 62 °C for 5 min, after which 145 μL water and 25 μL 10% Triton X-100 were added and incubated at 37 °C for 15 min. Next, the nuclei in each tube were digested by adding 25 μL 10x NEB buffer 3 (100 mM NaCl, 50 mM Tris-HCl, 10 mM MgCl_2_, 1 mM DTT, pH 7.9) and 50 U of DpnII restriction enzyme and incubated at 37 °C overnight. The next day, the nuclei were incubated at 62 °C for 20 min to inactivate the restriction enzyme. Next, the digested chromatin was blunt-ended by adding 1 μL of 10 mM dTTP, 1 μL of 10 mM dATP, 1 μL of 10 mM dGTP, 25 μL of 0.4 mM biotin-14-dCTP, 14 μL water, and 4 μL (40 U), Klenow fragment and incubated at 37 °C for 2 h. Subsequently, 663 μL water, 120 μL 10x blunt-end ligation buffer (300 mM Tris-HCl, 100 mM MgCl_2_, 100 mM DTT, 1 mM ATP, pH 7.8), 100 μL 10% Triton X-100, and 20 Weiss U T4 DNA ligase were added to start proximity ligation. The ligation reaction was placed at room temperature for 4 h. After ligation, the nuclei were collected by spinning them down at 1000 rcf for 3 min and then resuspended in 750 μL SDS buffer (50 mM Tris-HCl, 1% SDS, 10 mM EDTA, pH 8.0) and incubated with 200 μg proteinase K at 55 °C for 30 min. The formaldehyde crosslink was reversed by adding 30 μL 5 M NaCl to the solution followed by overnight incubation at 65 °C. The recovery of Hi-C DNA and subsequent DNA manipulations were performed as described [75]. The final library was sequenced on an Illumina HiSeq 3000 instrument with 2 × 150 bp reads.

### Hi-C read mapping and filtering

Hi-C reads from both hybrid and parents were mapped to the synthetic hybrid genome assembly as described above for RNA-seq. With an iterative mapping strategy [66], the 5’ 100 bp sequences of reads 1 and 2 of the mate pairs were mapped independently and only paired-end reads with both mates uniquely mapped were kept. Removal of polymerase chain reaction (PCR) duplicates and read filtering were performed as described [66], except that the hybrid reference genome was used. Hi-C reads from each biological replicate used in this study are summarized in Additional file 4: Table S3.

### Hi-C map normalization

For normalization, a count matrix was firstly created and filled with all filtered Hi-C reads with respect to their genomic positions. An iterative bias correction method was employed to account for technical biases, such as PCR amplification efficiency, restriction sites density, and mapping biases [104]. Under an assumption that each bin should have equal “visibility” (sequence coverage) in a Hi-C experiment, the bias correction process adjusted the count matrix so that at the end each row or column of the Hi-C matrix had similar sum values [105]. To this end, an iterative matrix correction function in the “HiTC” package in R was used [106]. We stopped the iterative correction loop when the *eps* value, which was used as a measurement of how similar the matrices in two consecutive correction steps were, dropped below 1 × 10^–4^. Normalization at 20-kb resolution was done at a genome-wide level (i.e. all chromosomes were included), while normalization at 5-kb resolution was done for each chromosome separately. In the 20-kb resolution Hi-C map of the hybrid, we observed signals of interactions between chromatin from the two parents that were highly correlated with the distribution of syntenic blocks of the two genomes [83] (Additional file 1: Figure S5C). These signals were not discussed in this study, as our analyses indicated that mapping errors were at least partially responsible for these patterns (Additional file 1: Figures S18 and S19). The overall impact of reads mapping errors on calculating interaction decay exponents was negligible (see below).

### Calculation of chromatin compactness and interaction decay exponents

To calculate chromatin compactness (Fig. 3e), the entries in a normalized Hi-C matrix (5-kb resolution) were first divided by the average value of entries, resulting in measurements of neighboring bin interactions. Such scaling procedure offset differences between Hi-C matrices due to different sequencing depths. For each 5-kb bin, its chromatin compactness was defined as the sum of its interactions with all bins located 10–50 kb away.

The interaction decay exponent of 100-kb genomic regions (Additional file 1: Figure S7) was calculated as follows: a subset of the normalized Hi-C matrix (5 kb) corresponding to a given 100-kb region was extracted and the average value of entries indicating chromatin interaction strengths of 5-kb, 10-kb, 15-kb, …, 50-kb distances was calculated accordingly. Because entries associated with bins masked in the Hi-C matrix normalization step were excluded, at the end, there might be fewer than ten average values. We only continued with regions that generated at least six average values, which we used subsequently for linear regression with the “lm” function in R. The resulting slope was defined as the interaction decay exponent.

Because both the *A. thaliana* and *A. lyrata* genomes showed a genome-wide even error rate in read mapping (Additional file 1: Figures S18B and S19B), during the calculation of the interaction decay exponents and after logarithm transformation, the error rate became a constant that only changed the y-axis intercept, but not its slope.

### Bisulfite sequencing and data analysis

Genomic DNA was extracted from leaves of hybrid and parent seedlings with the DNAeasy Plant Mini Kit (Qiagen). DNA was sheared to 350 bp with a Covaris™ S220 sonicator. DNA end repair, A-tailing, and adaptor ligation were performed with a TruSeq Nano Kit (Illumina) according to the manufacturer’s instructions. After adaptor ligation, the bisulfite treatment was done with an Epitect Plus DNA Bisulfite Conversion Kit (Qiagen). PCR amplification of library molecules was done using KAPA Hifi Uracil + DNA Polymerase (Kapa Biosystems). Libraries were sequenced on an Illumina HiSeq 3000 instrument with 2 × 150 bp reads.

All paired-end reads were aligned to the synthetic hybrid reference genome using Bismark (v0.15.0) [107] with Bowtie 2 aligner (v2.2.4) [108] with default parameter settings. PCR duplicates were removed after mapping. The unmethylated and methylated cytosine residue(s) in every read were identified in all sequence contexts (CG, CHG, and CHH) by the “bismark_methylation_extractor” script in Bismark. The methylation ratio of a given genomic region was calculated as the ratio between the total number of identified 5-methylcytosines and the total number of sequenced cytosines.

### ChIP-seq and data analysis

Tissue fixation and nuclei extraction were performed according to [75] and nuclei from 1 g of seedlings were used for one round of ChIP. The ChIP experiments essentially followed [66] with minor changes. In brief, chromatin was sheared to an average size of 350 bp with a Covaris E220evolution™ sonicator. The sonicated sample was halved and immunoprecipitated with 2 μg of anti-H3 (Abcam ab1791) or 2 μg of anti-H3K27me3 antibodies (Millipore, 07-449). After overnight incubation at 4 °C, the antibodies were recovered with 15 μL Protein A/G magnetic beads (Pierce) followed by a series of washing steps as described [66]. The ChIP-ed DNA was extracted with a standard phenol-chloroform method and the subsequent end repairing, A-tailing, adaptor ligation, and library amplification steps were performed with the NEBNext® Ultra™ II DNA Library Prep Kit (NEB). The library was sequenced on an Illumina HiSeq 3000 instrument with 2 × 150 bp reads.

Reads were aligned against the synthetic hybrid reference genome, as described above, using Bowtie 2 v2.2.4 [108] with mapping parameters as “-R 5 -N 0 -L 20 -i S,1,0.50.” The mapped reads were analyzed by MACS2 v2.1.0.20140616 [109]. The “--broad” flag was on during peak calling, with reads from the anti-H3 sample used as control and default settings were used for the other parameters.

### FISH

FISH probes were labeled with digoxigenin-11-dUTP by nick translation. A collection of bacterial artificial chromosome (BAC) cloning containing *A. thaliana* genomic fragments belonging to a 5.3-Mb interval on the right arm of chromosome 1 were used (Additional file 6: Table S5). The final probe concentration was 50 ng/μL.

Nuclei were isolated from fixed plant tissue, stained with DAPI, and sorted with a MoFlo™ XDP FACS instrument (Beckman Coulter) using a 100-μm CytoNozzle and standard PBS as sheath. Nuclei were sorted in Purify 1-drop mode. DAPI was excited using an OBIS 375 nm LX laser at 40 mW focused using a Near UV achromatic lens (Newport PAC14AR.15) with scatter collected from a 488-nm Argon (70 mW) and elliptically focused. Peak DAPI was collected in FL8 (405/30) and FL9 (465/30), with the shoulder in FL10 (542/27). Events were triggered off FL8 and nuclei were first identified by SSC-LA versus FL9-LA. Clumps were removed by sequential plotting of SSC-W, FSC-W, FL8-W versus FL8-LH with further auto-fluorescent debris removed by plotting FL8-LH versus FL10-LH. Finally, fully gated nuclei with different ploidy levels were inferred according to their increasing DAPI signal intensities in a bivariate FL8-LH versus SSC-LA plot; only the 2C nuclei were collected. The nuclei were then transferred onto a glass slide. Next, the pre-hybridization, hybridization, post-hybridization wash, and probe detection steps were performed as described with minor modifications [110, 111], with hybridization carried out at 45 °C for 20 h. After the final wash step, slides were mounted with SlowFade® Diamond Antifade Mountant with DAPI (Thermo Fisher Scientific).

### Fluorescence microscopy and image processing

Confocal images were acquired with a Zeiss LSM 880 Airyscan system. For each nucleus, z-stack images (with 0.22 μm thickness for each optical sectioning) were taken with a 63× objective lens. The detection of DAPI and Alexa Fluor® 488 was according to the following settings: laser power = 1.3% for 405 nm and 0.67% for 488 nm; pinhole = 1 AU; filter = BP 420–480 + BP 495–550; master gain = 700; digital gain = 2. Same parameter settings were applied to all types of nuclei for image acquisition. Because each *A. thaliana* nucleus has two copies of target genomic DNA, it is not possible to determine the volume occupied by each copy if they overlap in space. Thus, for ease of image processing, we only recorded images of *A. thaliana* nuclei displaying two distinct clusters of FISH signals. Image processing was done with Fiji software and images were finally assembled in Photoshop. The volumes occupied by the probed genomic region in different nuclei were approximated according to the sum of areas of filtered FISH signals in z-stack images (Additional file 1: Figure S20). To identify pixels with FISH signal in each z-stack image, a threshold value of 25 in the green channel was used throughout.

## Additional files

Additional file 1: Figures S1–S20. Supplemental references and Supplemental **Figures S1–S20**. (DOCX 13462 kb)

Additional file 2: Table S1. RNA-seq analysis, including reads counting, statistical analysis, on gene expression changes. (XLSX 11493 kb)

Additional file 3: Table S2. GO analysis of differentially expressed genes. (XLSX 82 kb)

Additional file 4: Table S3. Statistics of Hi-C reads. (XLSX 9 kb)

Additional file 5: Table S4. Genes enriched for H3K27me3 marks. (XLSX 3496 kb)

Additional file 6: Table S5. BAC vectors used for making FISH probes. (XLSX 44 kb)
