# Supplementary material for: Altered chromatin compaction and histone methylation drive non-additive gene expression in an interspecific Arabidopsis hybrid
Source: Genome Biol. 2017 Aug 22;18:157. doi: 10.1186/s13059-017-1281-4 (PMC5568265; doi:10.1186/s13059-017-1281-4)
Supplement: Supplementary file 1 — Supplemental references and Supplemental Figures S1–S20. (DOCX 13462 kb) [file 13059_2017_1281_MOESM1_ESM.docx]

**Altered chromatin compaction and histone methylation drive non-additive gene expression in an interspecific *Arabidopsis* hybrid**

Wangsheng Zhu, Bo Hu, Claude Becker, Ezgi Süheyla Doğan, Kenneth Wayne Berendzen, Detlef Weigel and Chang Liu

Supplemental References

1. Stroud H, Greenberg MV, Feng S, Bernatavichute YV, Jacobsen SE: Comprehensive analysis of silencing mutants reveals complex regulation of the Arabidopsis methylome**.** *Cell* 2013, **152:**352-364.

2. Stroud H, Do T, Du J, Zhong X, Feng S, Johnson L, Patel DJ, Jacobsen SE: Non-CG methylation patterns shape the epigenetic landscape in Arabidopsis**.** *Nat Struct Mol Biol* 2014, **21:**64-72.

3. Grob S, Schmid MW, Grossniklaus U: Hi-C analysis in Arabidopsis identifies the KNOT, a structure with similarities to the flamenco locus of Drosophila**.** *Mol Cell* 2014, **55:**678-693.

4. Liu C, Wang C, Wang G, Becker C, Zaidem M, Weigel D: Genome-wide analysis of chromatin packing in Arabidopsis thaliana at single-gene resolution**.** *Genome Res* 2016, **26:**1057-1068.

**
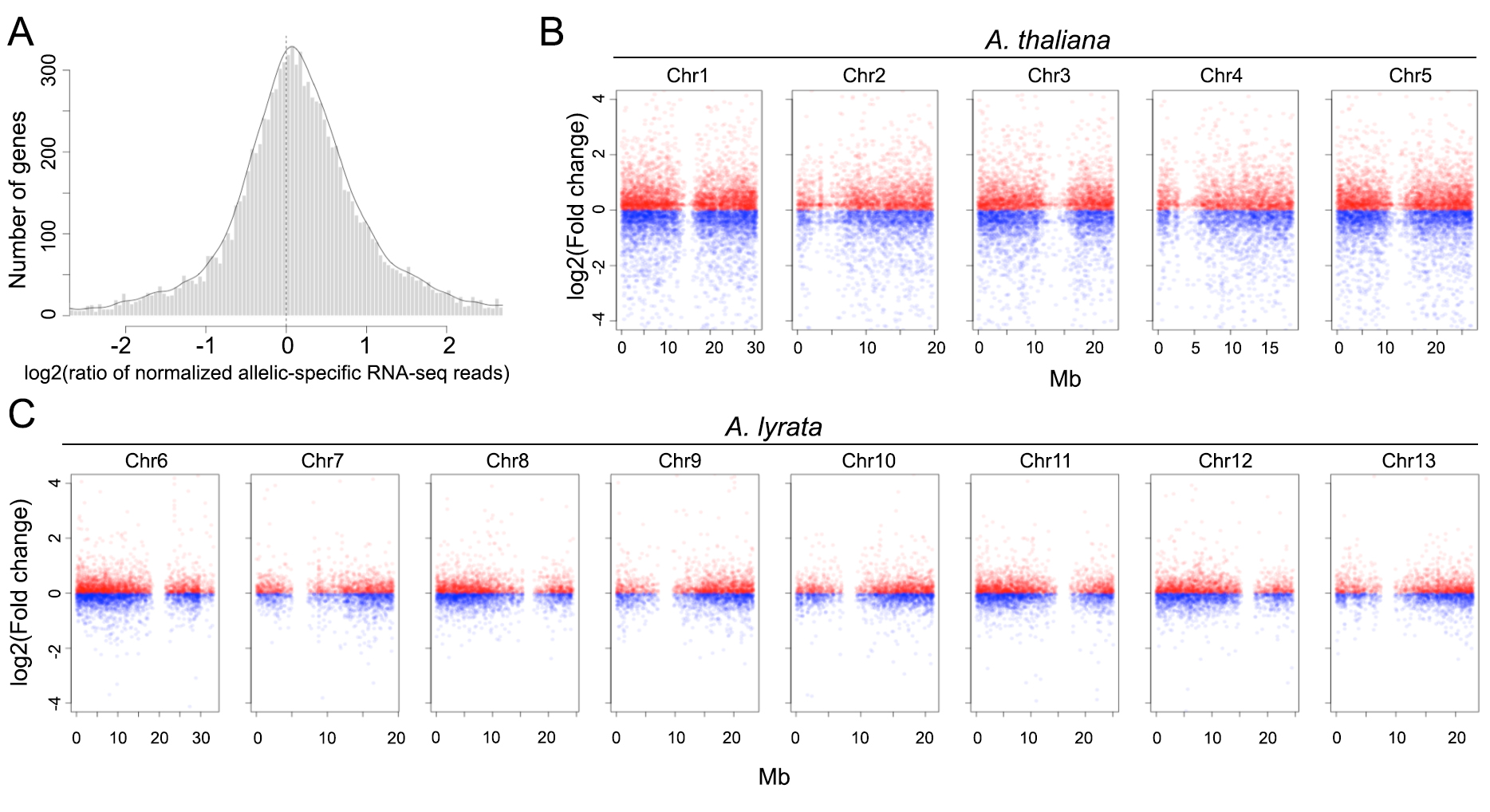
**

**Figure S1. Changes of gene expression and allele bias in the hybrid** (A) Distribution of allelic gene expression ratios (*A. lyrata* over *A. thaliana*) in the hybrid. For a pair of allelic genes, the number of reads mapped uniquely to each allele was firstly normalized by annotated transcript length, and the ratio of expression level of the two genes was obtained by dividing the value of the *A. lyrata* gene by that of the *A. thaliana* gene*.* (B and C) Gene expression ratios of hybrid over parent along each chromosome. Red and blue colors mean positive and negative log-ratios, respectively. The *A. lyrata* chromosomes 1 to 8 were re-named as chromosomes 6 to 13, respectively.

**
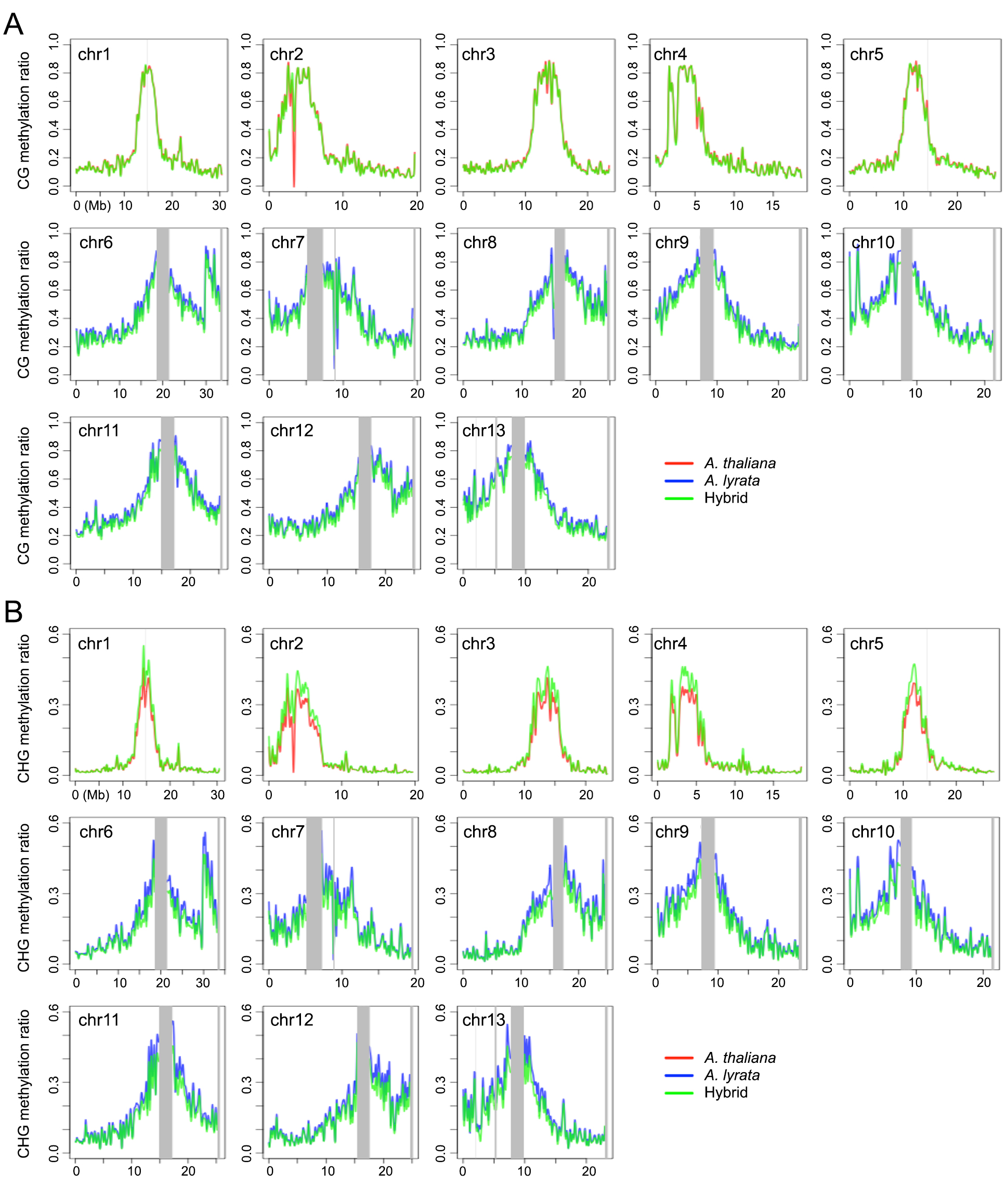
**

**Figure S2. DNA methylation in the parent and hybrid plants** DNA methylation levels in CG (A) and CHG (B) contexts along chromosomes. Labels as Figure 2A.

**
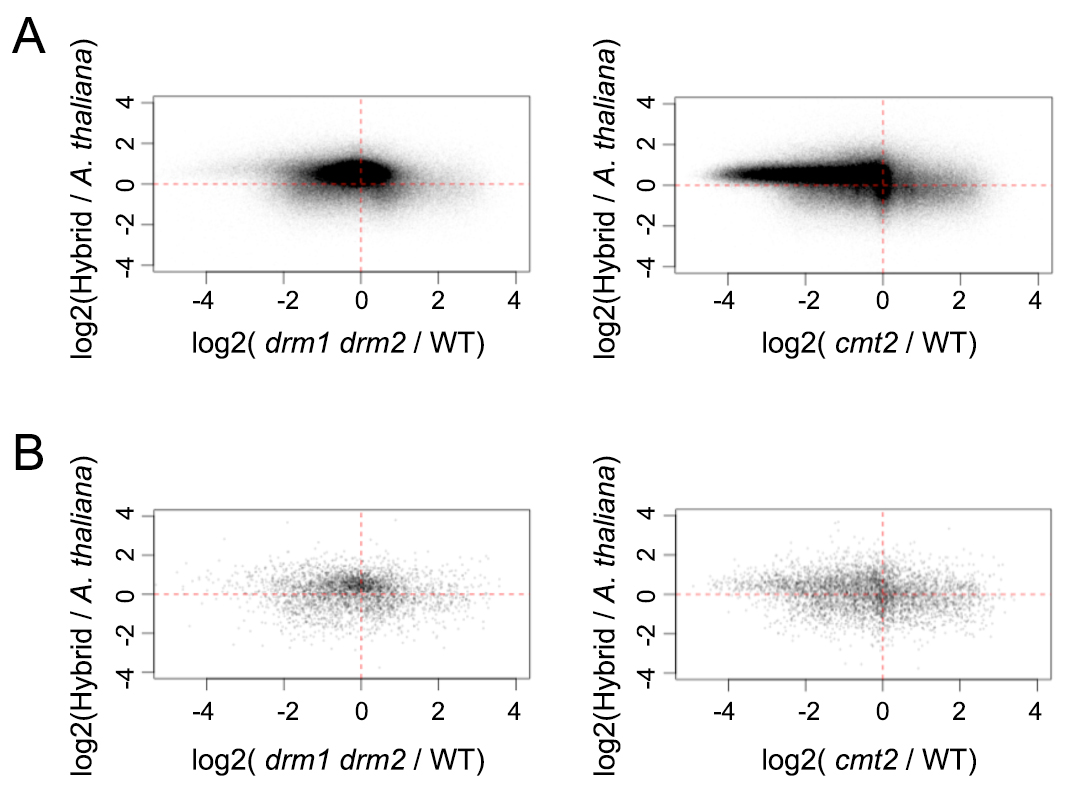
Figure S3. Changes of *A. thaliana* CHH methylation in the hybrid and two *A. thaliana* DNA methylation mutants** (A) Distributions of genome-wide changes in CHH methylation ratios of hybrid over *A. thaliana* wild type (y-axis) or mutant over wild type (WT) (x-axis) [1, 2]. Ratios were calculated in 100 bp windows. (B) Same as (A), but only including genomic regions fulfilling the following criteria: 1) located at least 2 Mb away from pericentromeres, and 2) CHH methylation ratios above 0.015 in the hybrid.

**
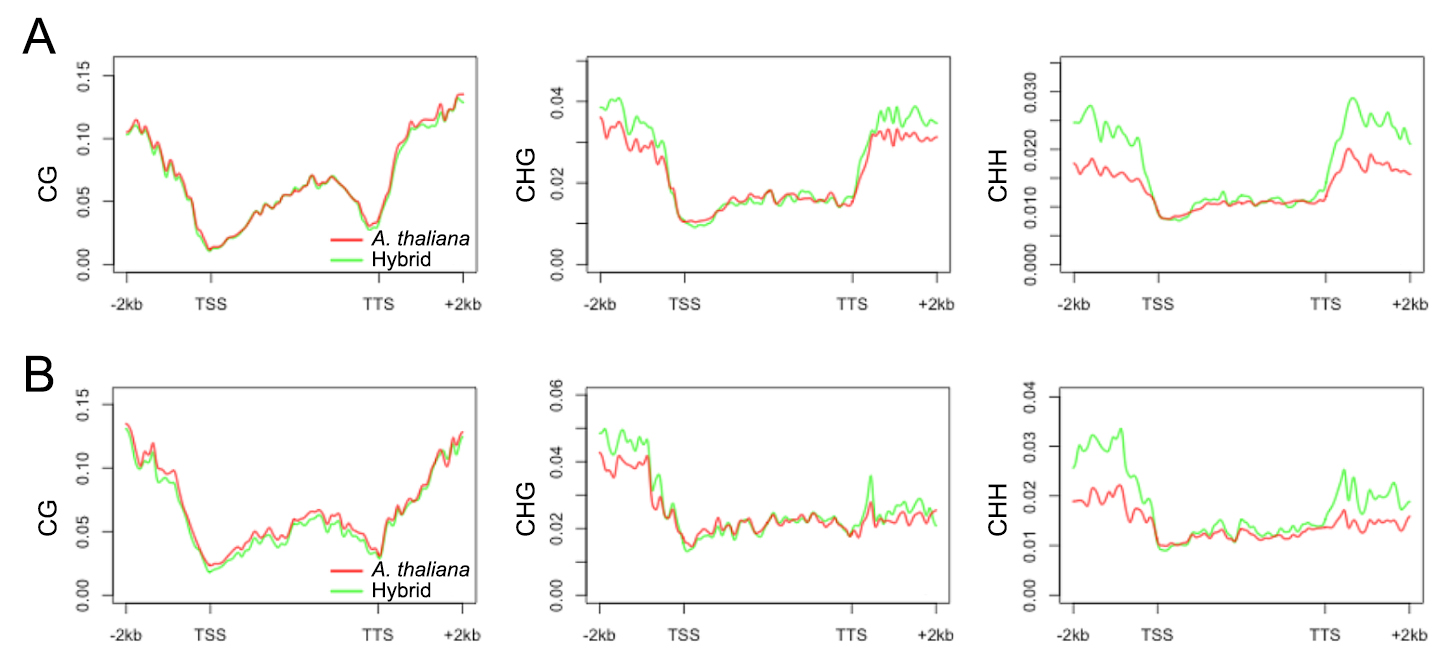
**

**Figure S4. DNA methylation on genes differentially expressed in hybrid** Each sequence context of DNA methylation was analyzed on *A. thaliana* genes that are down-regulated (A) or up-regulated (B) in the hybrid. Labels as in Figure 2D.


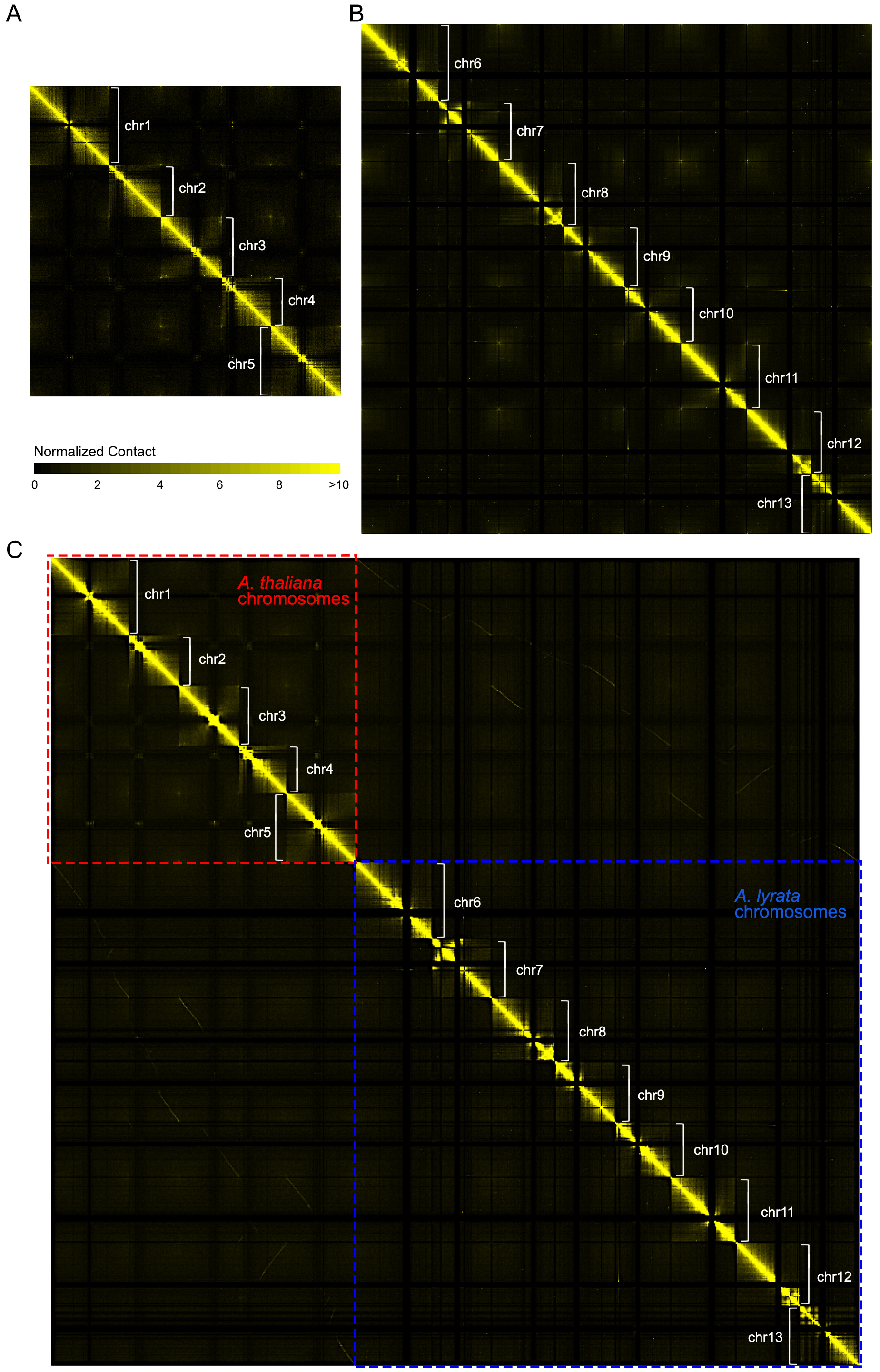
**Figure S5. Chromosome packing in hybrid and parents** Genome-wide Hi-C maps of *A. thaliana* (A), *A. lyrata* (B) and the hybrid (C) at 20 kb resolution.

**Figure S6. Hi-C read distribution as a function of chromatin interaction distance.** (A and B) Comparison of intra-chromosomal Hi-C reads
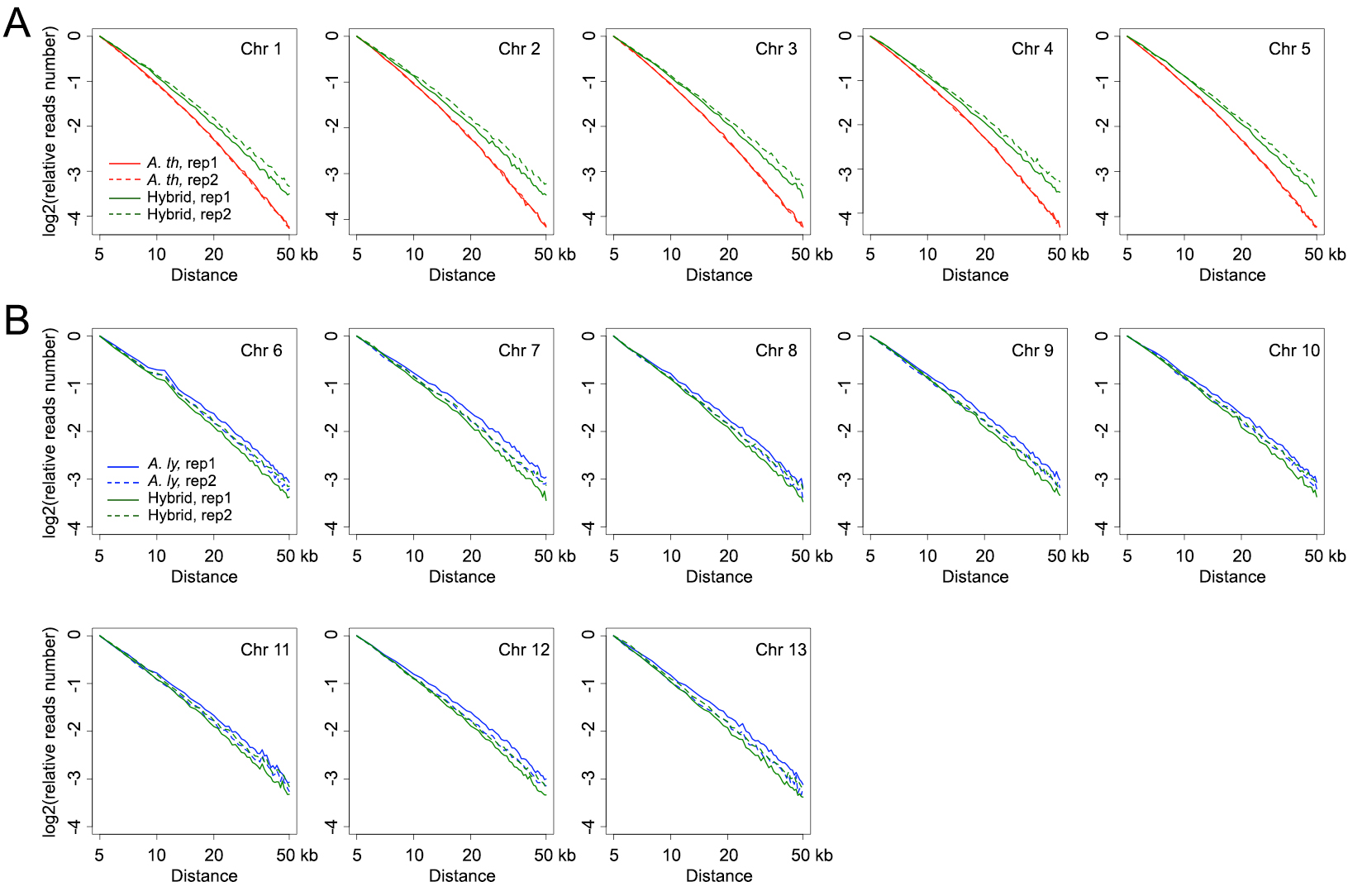
mapped to *A. thaliana* (A) and *A. lyrata* (B) chromosomes. Only Hi-C reads of interactions between 5 and 50 kb were included. These reads were sorted into 46 groups referring to distances of {5, 6, 7, ... 49, 50} kb, and normalized to read numbers found at 5 kb distance.

**
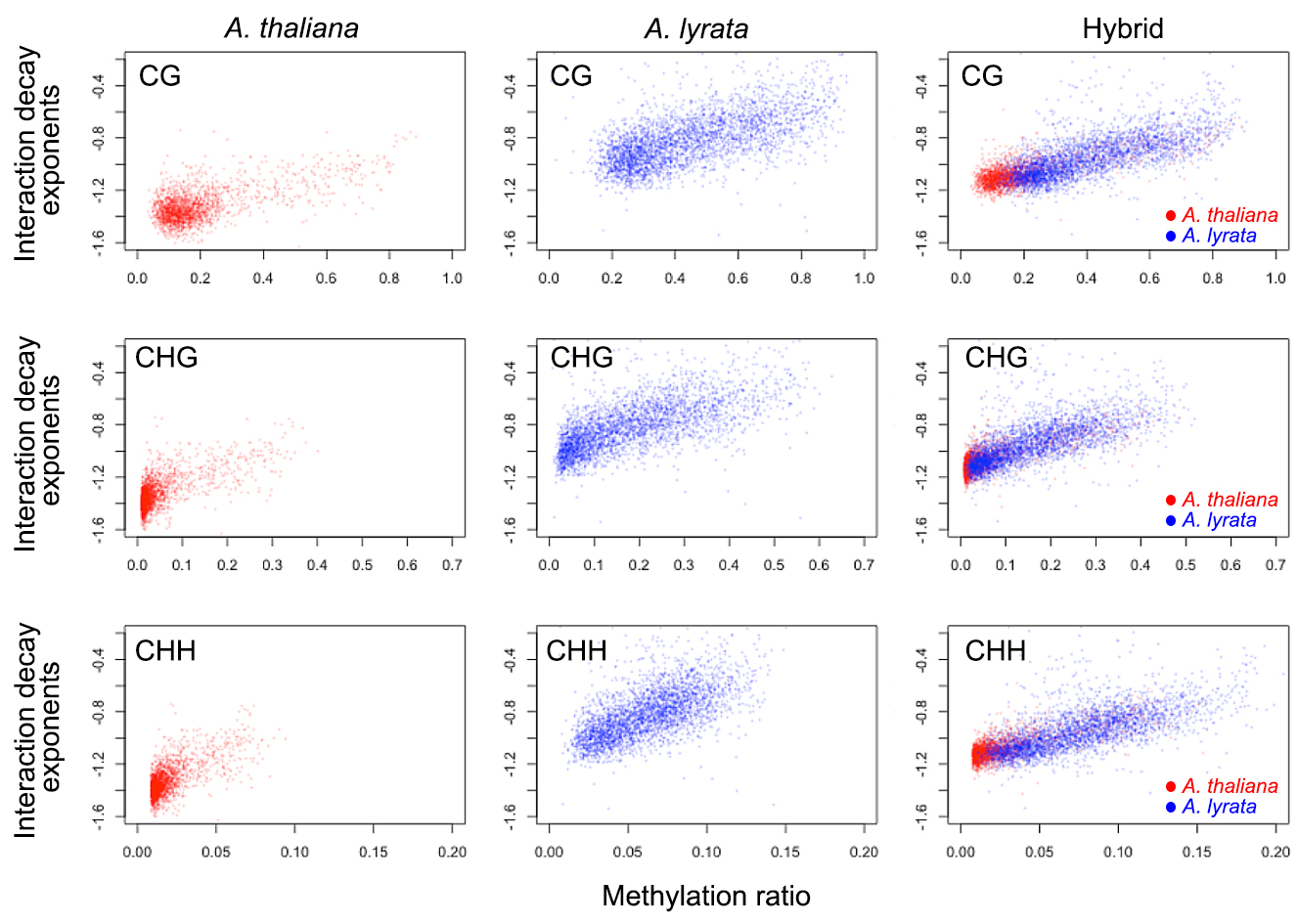
**

**Figure S7. Association between interaction decay exponents and DNA methylation**

Genomes were divided into 100 kb sliding windows with a 50 kb step size. In every plot, each point represents a 100 kb region, from which its interaction decay exponents and DNA methylation in various sequence contexts were calculated. For the calculation of interaction decay exponents, only interactions within a given 100 kb region and between 5 and 50 kb were included; see Methods for more details. For the hybrid, genomic regions corresponding to the parents are colored accordingly. Regions belonging to pericentromeres in the *A. thaliana* genome are excluded.

**
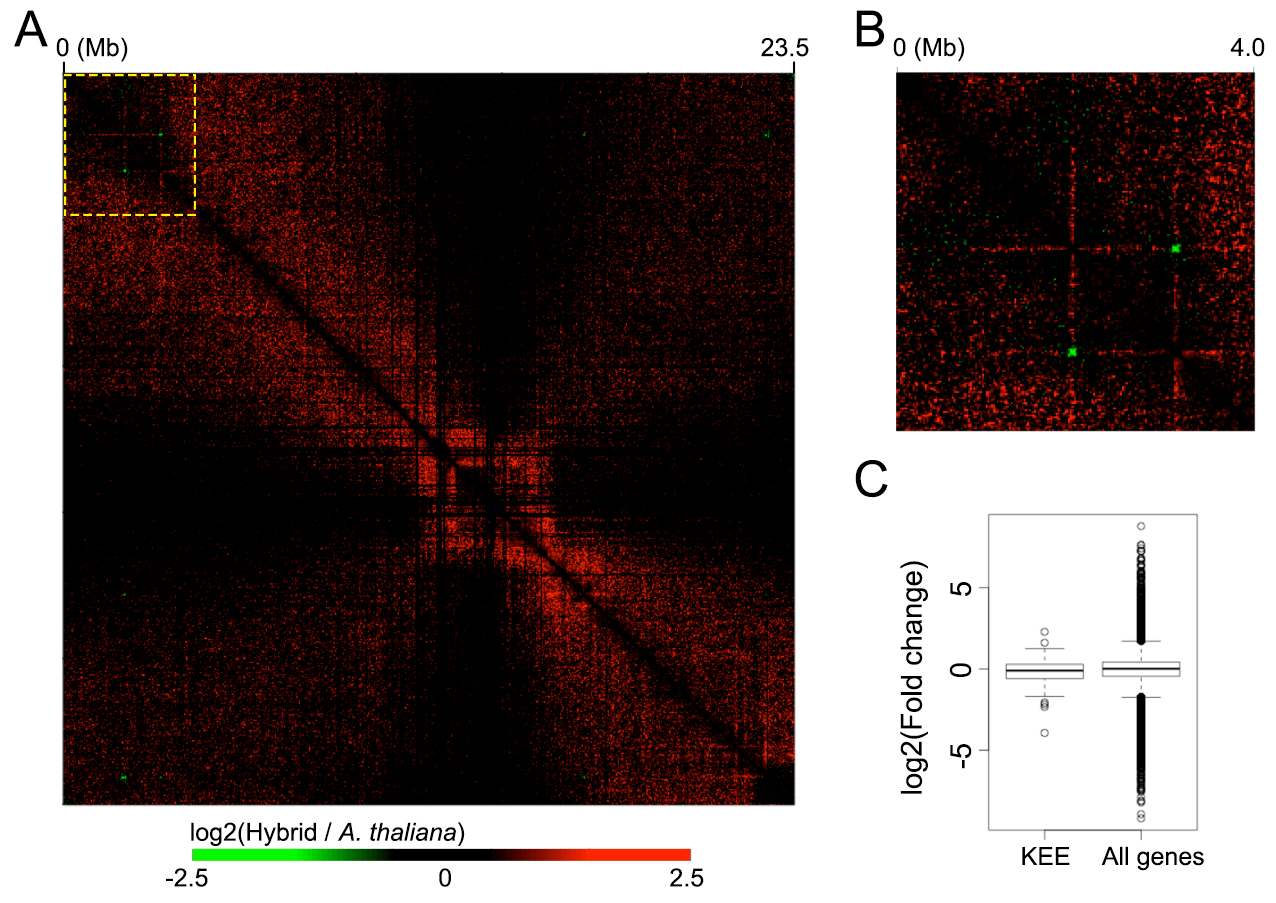
Figure S8. Effect of interspecific hybridization on chromosome organization** (A and B) Comparison of chromosome 3 Hi-C maps between the hybrid and *A. thaliana* at 20 kb resolution. Colors indicate the difference of chromatin interaction strengths, expressed as the ratio between hybrid and *A. thaliana* Hi-C maps. (B) Close-up of the telomere-proximal 4 Mb region, highlighted in (A) with yellow dashed borders. Green dots correlate with interactions between regions annotated as KEE [3]. (C) Gene expression ratios of hybrid over parent in KEE regions [3] compared to those of all genes. This comparison has a *p*-value above 0.05 from a Wilcoxon-Mann-Whitney test.


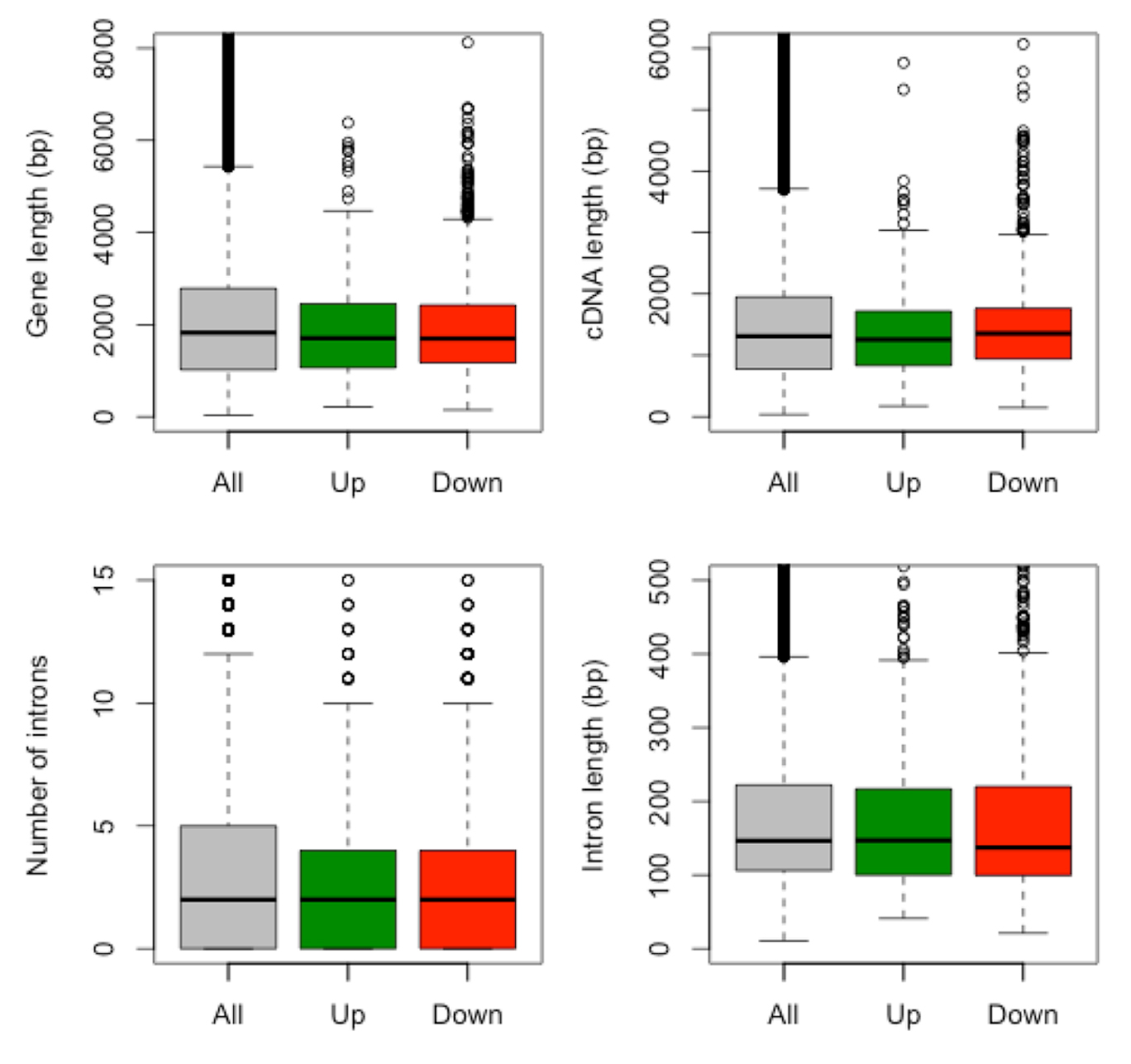
**Figure S9. Structural properties of differentially regulated genes** All pairs of comparisons using all genes as the background set have p-values above 0.05 from Wilcoxon-Mann-Whitney tests.

**
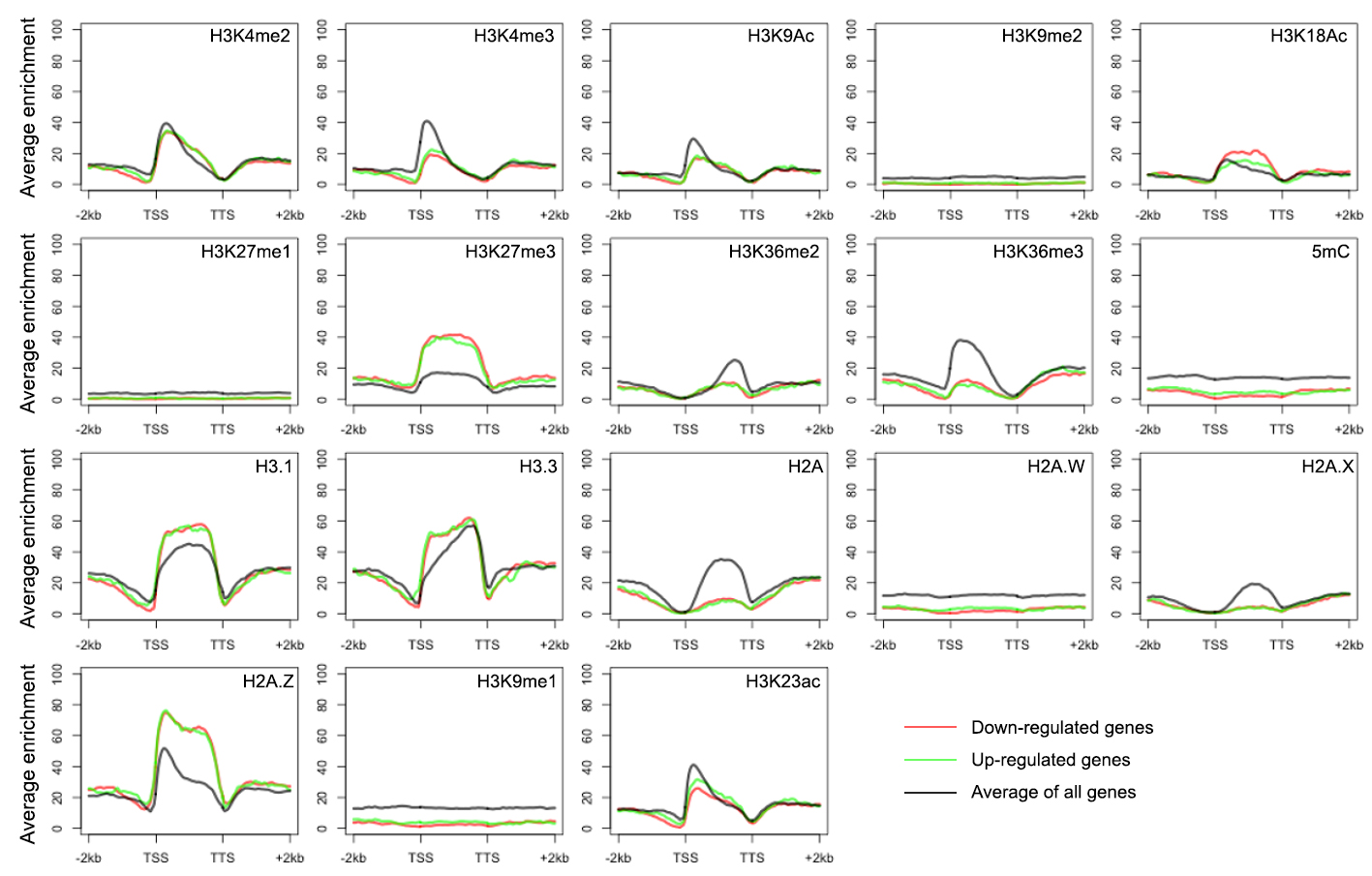
**

**Figure S10. Parental epigenetic marks around differentially expressed *A. thaliana* genes** The plots show the average of different histone modifications and histone variants in *A. thaliana* seedlings. Up- and down-regulated genes refer to differentially expressed *A. thaliana* genes in the hybrid. Genes are scaled to align their TSSs and TTSs. Average enrichment means the percentage of regions enriched for the respective epigenetic mark. The integrated data of the *A. thaliana* seedling epigenome at 100 bp bin size are from [4].

**Figure S11. Comparison of H3K27me3 in the hybrids and parents** Genome-wide H3K27me3 signals
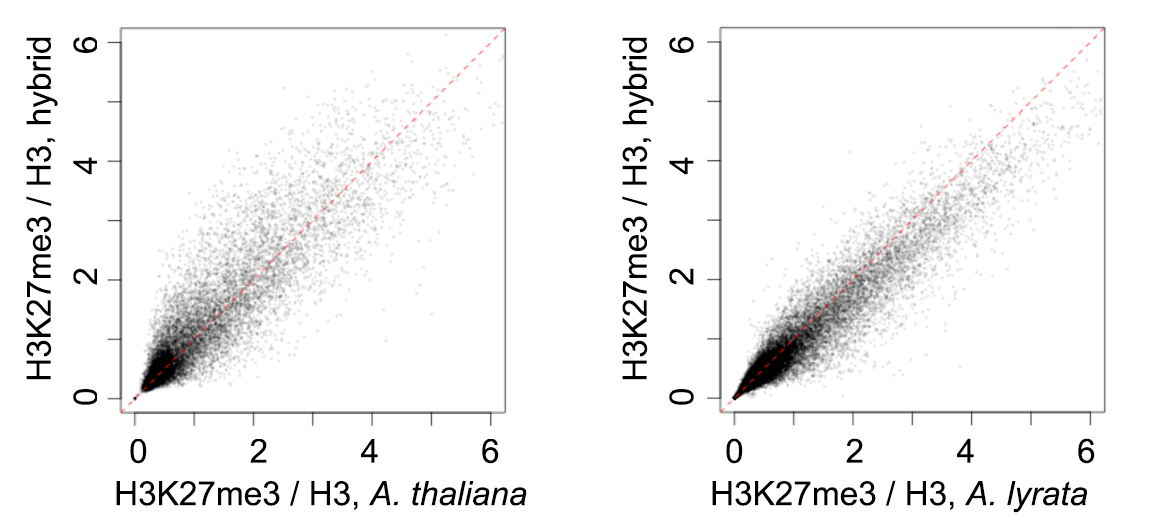
of *A. thaliana* (left) and *A. lyrata* (right) chromatin calculated in 5 kb windows.


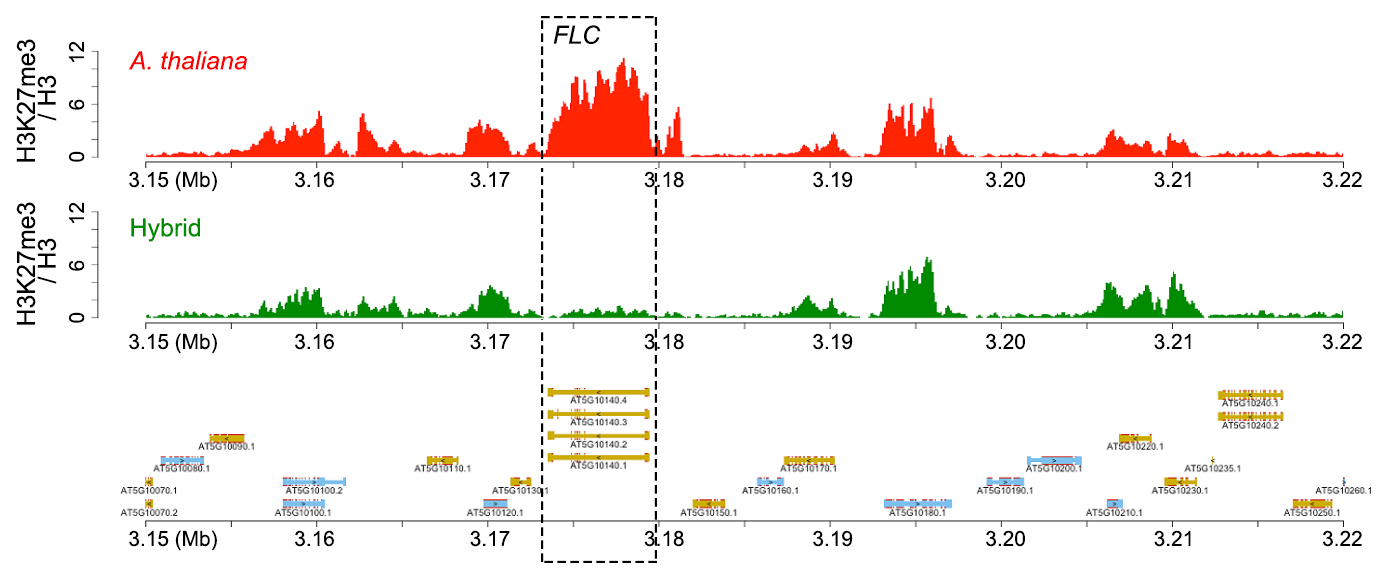
**Figure S12. Changes of H3K27me3 at the *FLC* locus** H3K27me3 ChIP-seq signals of a 70 kb region around *FLC* on *A. thaliana* chromosome 5. The bottom panel shows annotated genes (including splice variants) in this region. Genes encoded on the Crick and Watson strand are colored in blue and yellow, respectively.


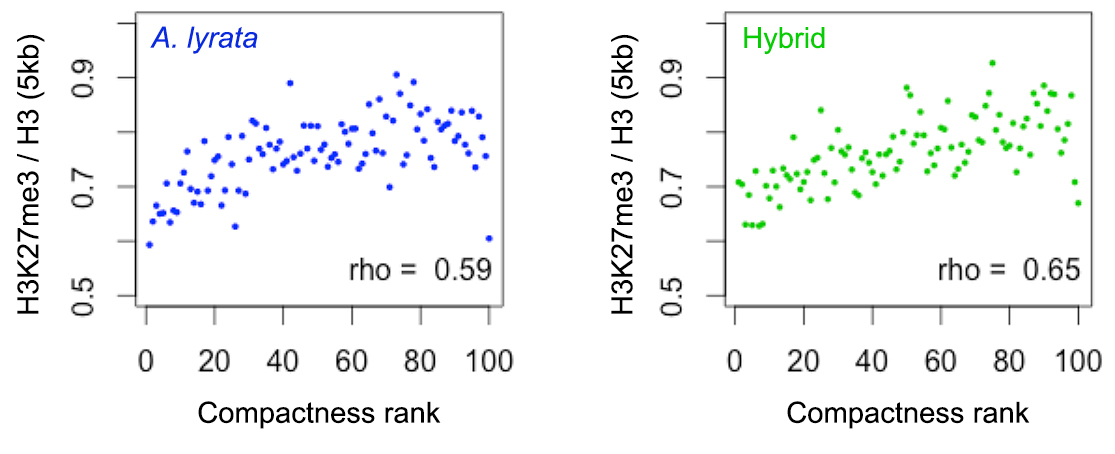
**Figure S13. *Arabidopsis lyrata* chromatin compactness and H3K27me3 signals** Average H3K27me3 signals in *A. lyrata* chromatin ranked from weakest to strongest chromatin compactness.


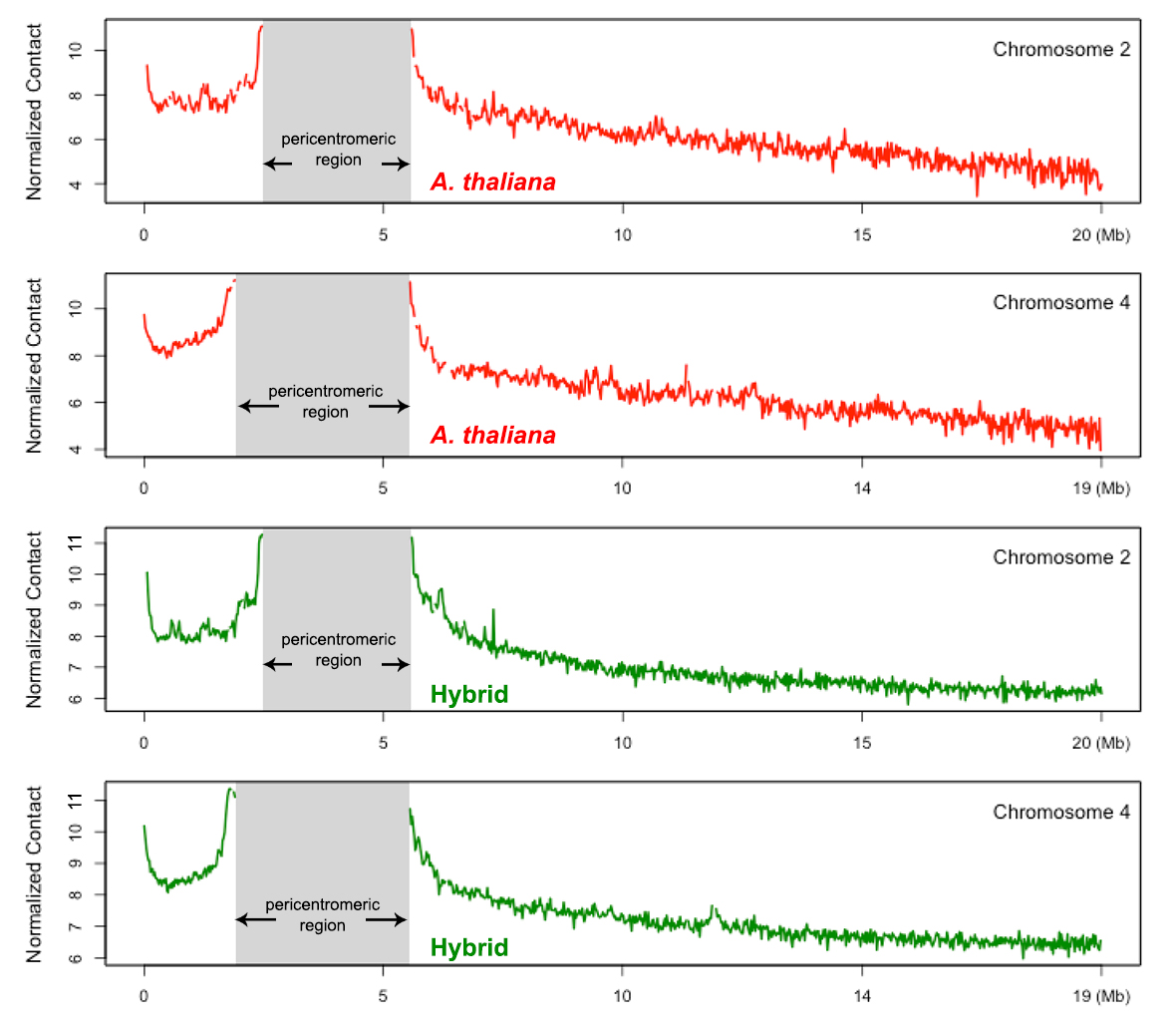


**Figure S14. Intra-chromosomal interactions between pericentromeric regions and chromosome arms** Calculations were performed with Hi-C maps normalized at 20 kb resolution. For each panel, bins belonging to pericentromeric regions (grey blocks) were used as query. The left ends of chromosome 2 and 4, which refer to the centromere-proximal boundary of *NOR2* and *NOR4*, interacted particularly strongly with pericentromeric regions.


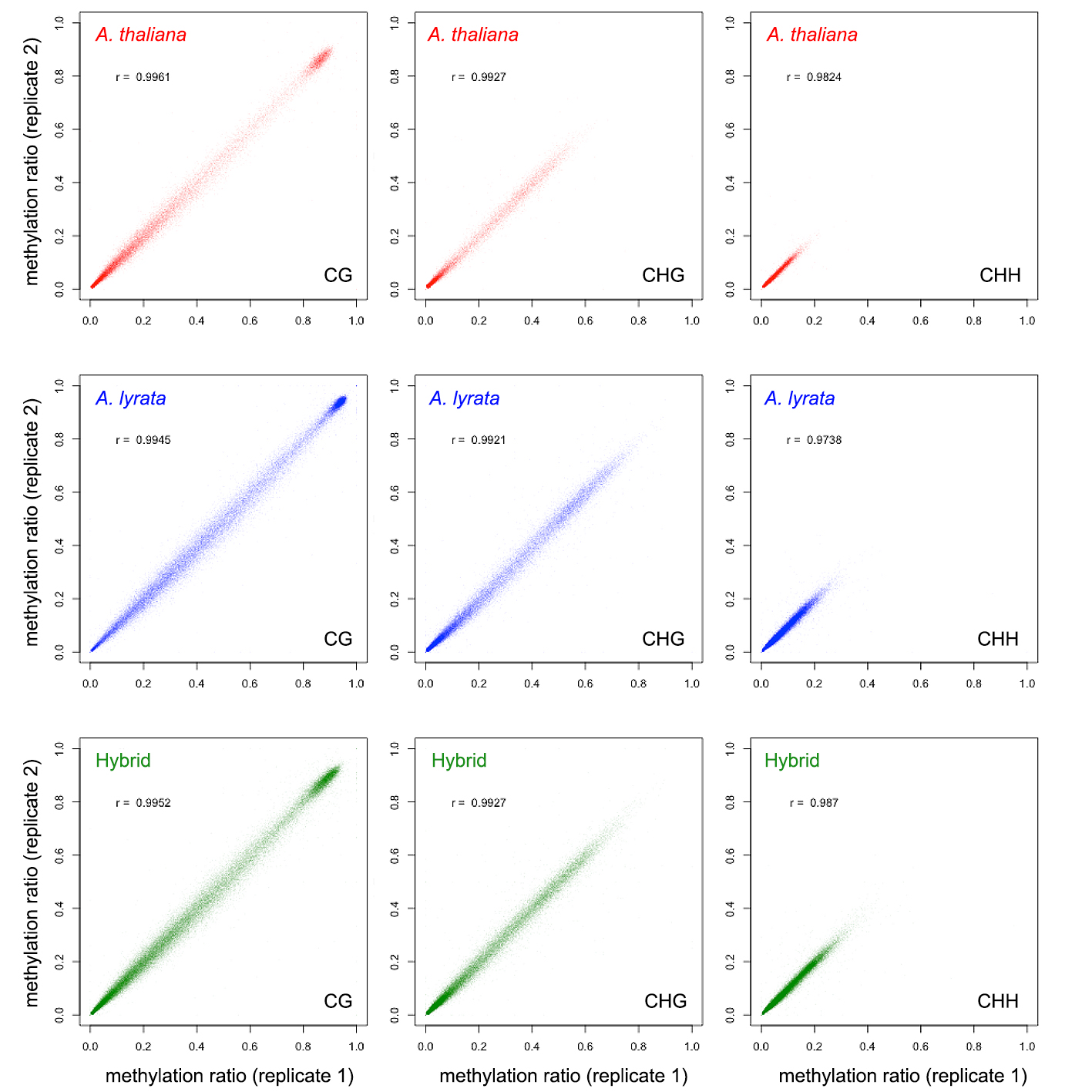


**Figure S15. Comparison of DNA methylation in replicates.** DNA methylation ratios were calculated in 5 kb windows. “r”, Pearson’s correlation coefficient.


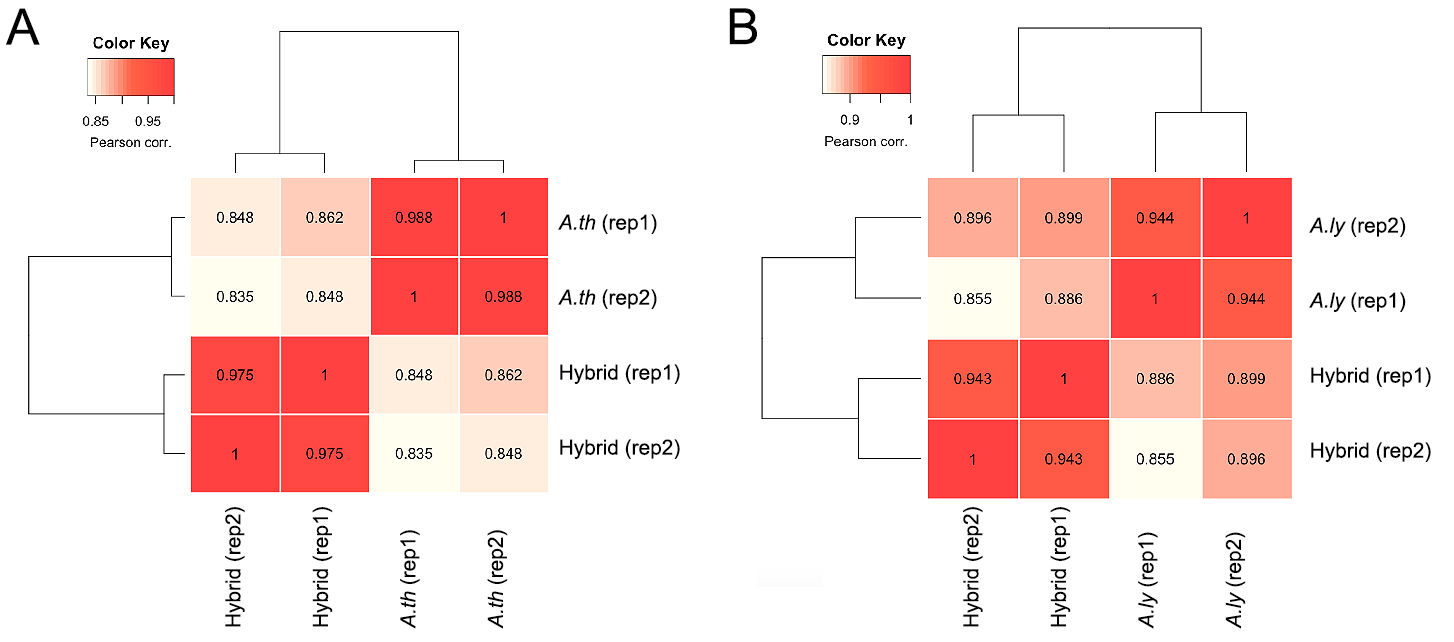


**Figure S16. Comparison of Hi-C maps.** Hi-C map (resolution: 20 kb) entries of intra-chromosomal interactions of the *A. thaliana* (A) and *A. lyrata* (B) chromosomes in the hybrid and parent. The dendrogram shows hierarchical clustering based on Euclidean distance.


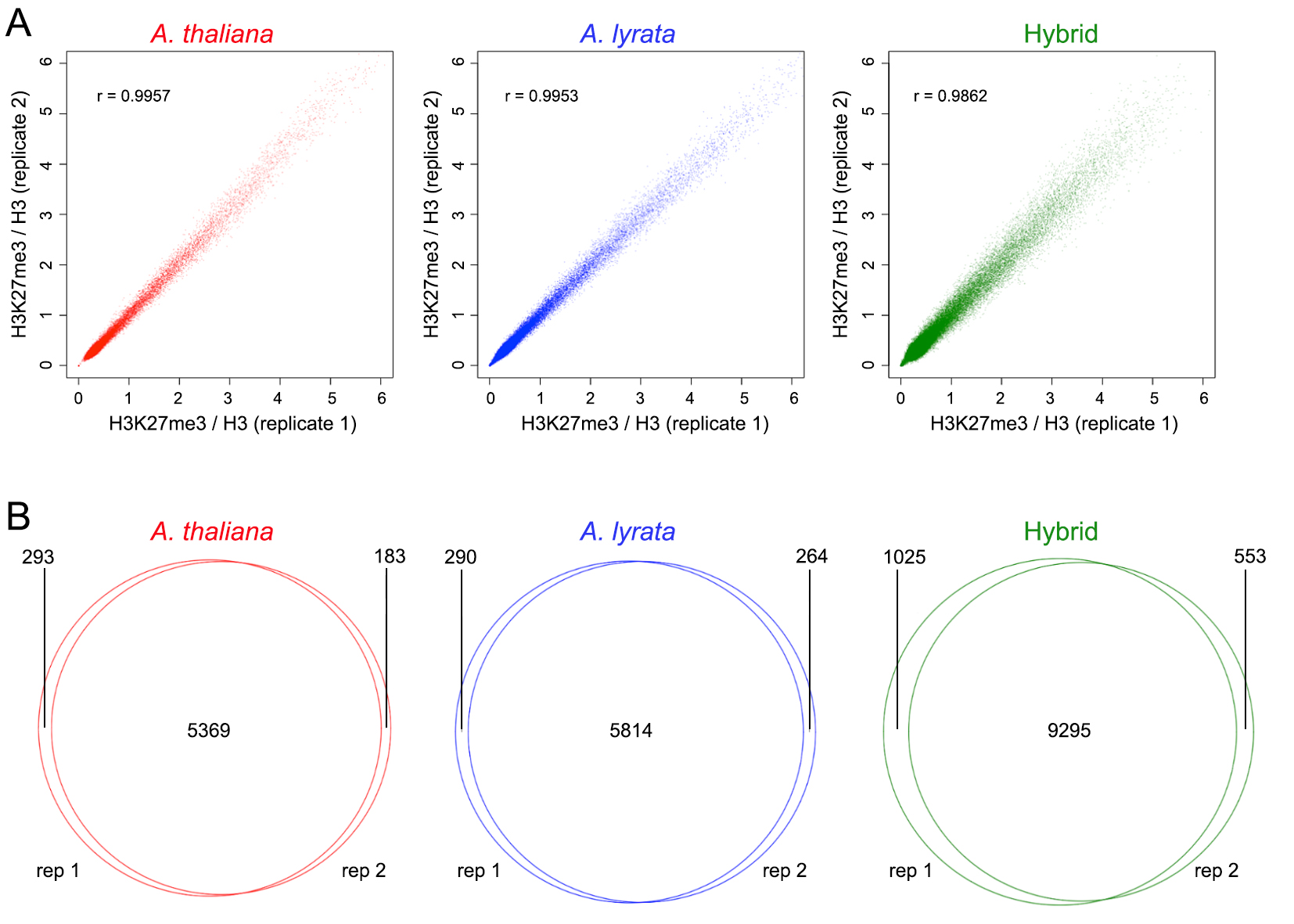


**Figure S17. Comparison of ChIP-seq replicates.** (A) ChIP-seq signals (H3K27me3 / H3) were calculated in 5 kb windows. “r”, Pearson’s correlation coefficient. (B) Venn diagram of genes enriched with H3K27me3. For each replicate, H3K27me3 peaks were called with the same set of parameters (described in Methods); a gene was considered enriched with H3K27me3 if more than half of its transcribed region overlaps with H3K27me3 enrichment peaks.


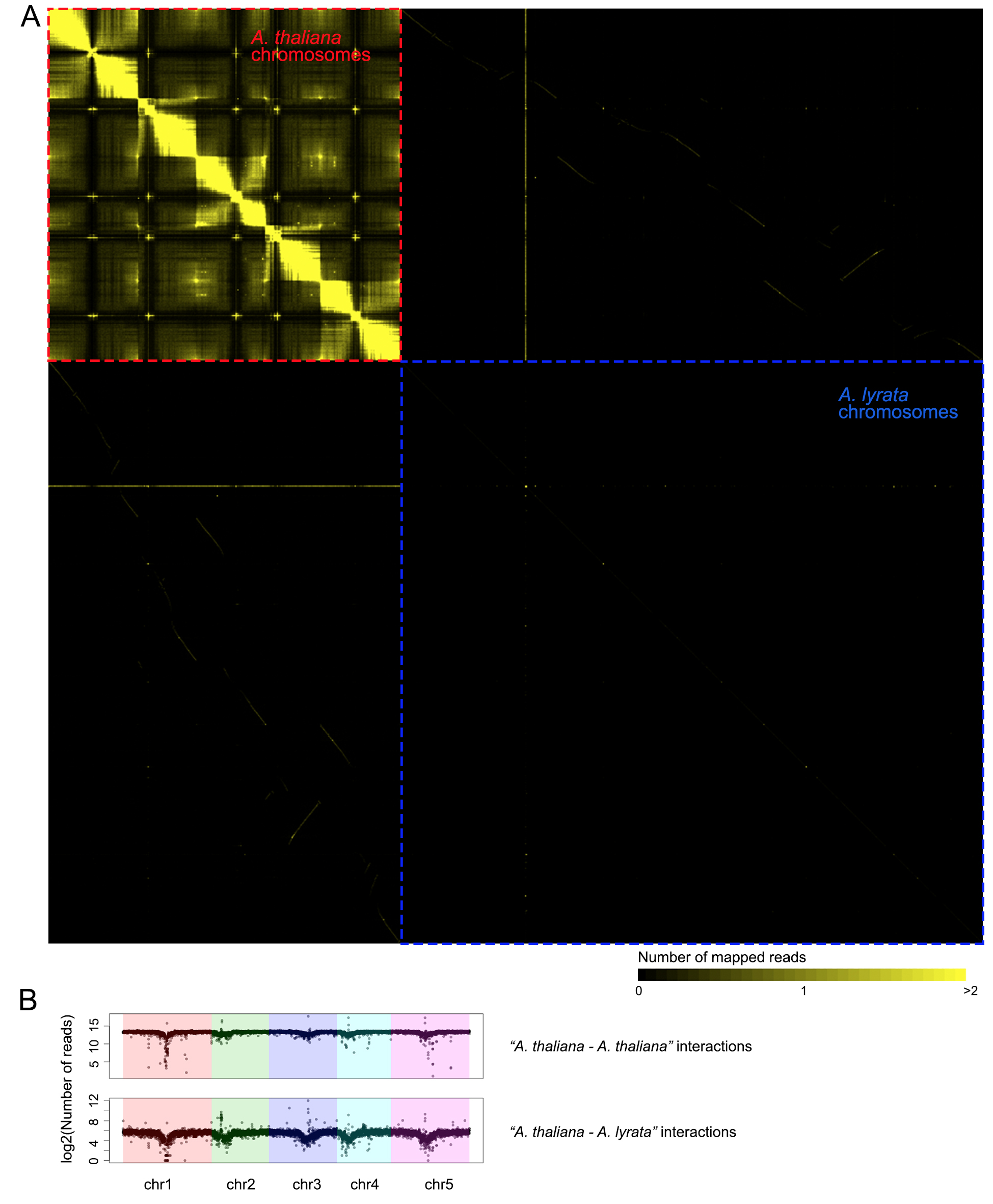


**Figure S18. Analysis of *A. thaliana*-onto-*A. lyrata* mapping errors.** (A) Distribution of mapped Hi-C reads originating from *A. thaliana* seedlings. The synthetic hybrid genome was used as mapping target (see Methods). Reads were assigned to 20 kb bins. (B) Distribution of reads that were erroneously mapped to the *A. lyrata* genome. Reads appeared as “*A. thaliana-A. thaliana*” and “*A. thaliana-A. lyrata*” interactions were collapsed to the *A. thaliana* bins, and the sum in each bin was calculated. Because most of the Hi-C reads resulted from short-distance intra-chromosomal interactions, it was assumed that for a given “*A. thaliana-A. lyrata*” interaction located in entry [*i, j*] (*i* and *j* referred to an *A. thaliana* and an *A. lyrata* bin, respectively), its correct entry was [*i, i*].


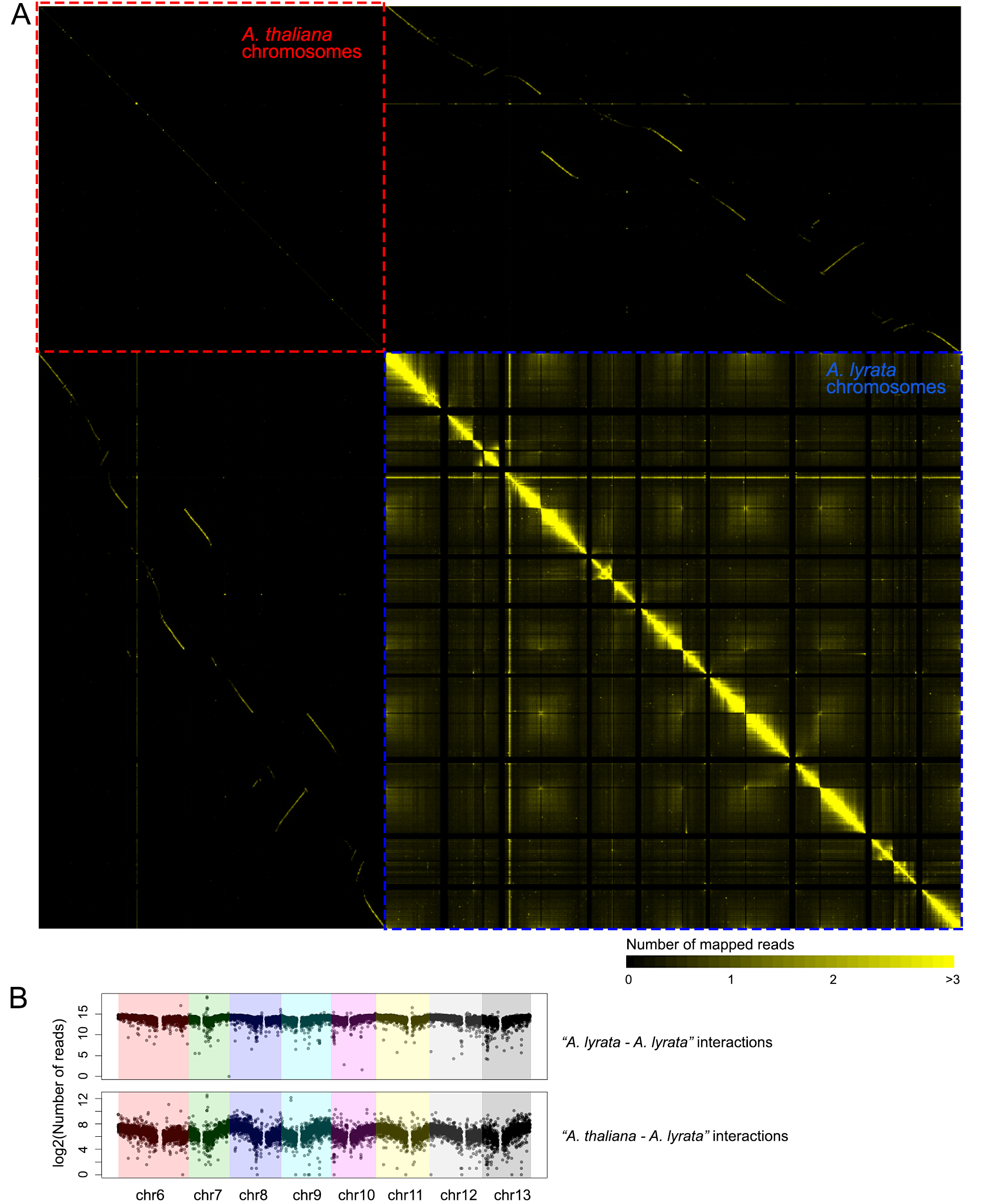


**Figure S19. Analysis of *A. lyrata*-onto-*A. thaliana* mapping errors.** (A) Distribution of mapped Hi-C reads originating from *A. lyrata* seedlings. The synthetic hybrid genome was used as mapping target (see Methods). Reads were assigned to 20 kb bins. (B) Distribution of reads that were erroneously mapped to the *A. thaliana* genome. Reads appeared as “*A. lyrata-A. lyrata*” and “*A. thaliana-A. lyrata*” interactions were collapsed to the *A. lyrata* bins, and the sum in each bin was calculated. Because most of the Hi-C reads resulted from short-distance intra-chromosomal interactions, it was assumed that for a given “*A. thaliana-A. lyrata*” interaction located in entry [*i, j*] (*i* and *j* referred to an *A. thaliana* and an *A. lyrata* bin, respectively), its correct entry was [*j, j*].

**
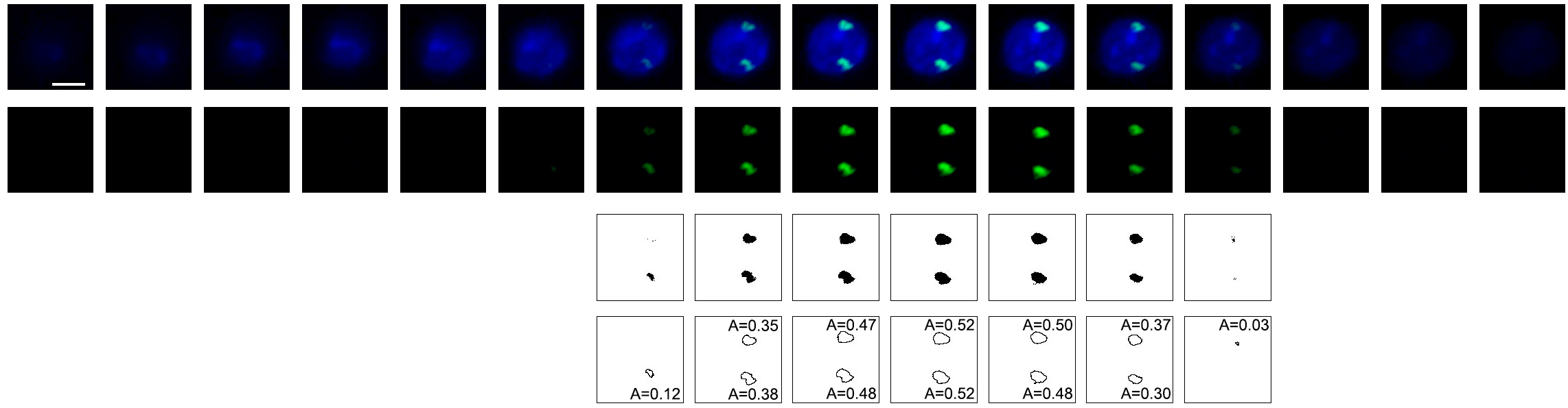
**

**Figure S20. Calculation of FISH signal volumes.** This example shows a nucleus from the *A. thaliana* parent. The first and second rows illustrate a series of z-stack (0.22 μm thickness for each optical sectioning) confocal images, where DNA (stained with DAPI) and FISH signals are shown in blue and green channels, respectively. Z-stack images that contain at least one pixel (marked in black) with FISH signal higher than the threshold value are shown in the third row. As the filtered pixels in each z-stack image always cluster, the area occupied by each pixel cluster is determined according to the cluster contour (the bottom row, unit in μm^2^, only clusters having areas larger than 0.01 μm^2^ are considered further). Finally, the volume of FISH signals in this nucleus is approximated as the sum of areas found in all z-stack images multiplied by the section thickness. In this example, signal volumes corresponding to the two genomic DNA copies are calculated separately. Scale bar, 2 μm.
